# Interrogation of macrophage-related prognostic signatures reveals a potential immune-mediated therapy strategy by histone deacetylase inhibition in glioma

**DOI:** 10.3389/fonc.2025.1554845

**Published:** 2025-06-06

**Authors:** Xisen Wang, Xuya Wang, Lei Chen, Haozhe Ding, Jikang Fan, Jianshen Liang, Yu Zhang, Yiming Li, Yiming Zhang, Shengping Yu, Chen Zhang, Yaohua Li, Tao Li, Xuejun Yang

**Affiliations:** ^1^ Department of Neurosurgery, Beijing Tsinghua Changgung Hospital, School of Clinical Medicine, Tsinghua University, Beijing, China; ^2^ Department of Neurosurgery, Tianjin Medical University General Hospital, Tianjin, China; ^3^ Department of Neurosurgery, Tianjin First Central Hospital, Tianjin, China; ^4^ Department of Neurosurgery, Tianjin Huanhu Hospital, Tianjin, China; ^5^ Laboratory of Neuro-Oncology, Tianjin Neurological Institute, Tianjin, China

**Keywords:** glioma, glioma-associated macrophages, prognostic signature, immune microenvironment, histone deacetylase inhibitors, vorinostat

## Abstract

**Background:**

Glioma-associated macrophages (GAMs) originate from intracranially resident microglia and myeloid-derived macrophages. In the glioma microenvironment, these two types of macrophages tend to adopt a specialized activation state known as type 2 or M2 macrophages and play crucial roles in the progression of glioma.

**Methods:**

To identify genes associated with GAMs, we intersected genes identified from single-cell RNA sequencing (scRNA-seq) data (specific to GAMs) with M2 macrophage module genes derived from weighted gene coexpression network analysis (WGCNA). Prognostic genes were screened using univariate Cox regression, multivariate Cox regression, and least absolute shrinkage and selection operator (LASSO) regression analysis. These genes were used to construct and validate prognostic signatures and to delineate the immune landscape. During drug screening, Vorinostat exhibited the highest risk score and the lowest half-maximal inhibitory concentration (IC_50_). The expression of the 14 prognostic genes was further investigated using a glioma cell-macrophage co-culture model.

**Results:**

Fourteen prognostic genes (TREM2, GAL3ST4, AP1B1, SLA, CYBB, CD53, SLC37A2, ABI3, RIN3, SCIN, SIGLEC10, C3, PLEKHO2, and PLXDC2) were identified. The prognostic model constructed from these genes demonstrated robust predictive efficacy. Based on this model, Vorinostat was prioritized as a candidate therapeutic agent, and subsequent validation confirmed its significant inhibitory effects on the glioma microenvironment.

**Conclusion:**

These findings elucidate the molecular mechanisms of GAMs in glioma, uncover the immunological landscape of the tumor microenvironment, and identify potential therapeutic targets and drug action mechanisms.

## 1 Introduction

Gliomas are the most common tumors of the central nervous system, among which glioblastoma (GBM) is the most malignant, accounting for 49.1% of all primary malignant tumors found in the central nervous system (1). The recent classification of central nervous system tumor by WHO in 2021 identified the integrated diagnosis of GBM as “Glioblastoma, IDH wild type, WHO grade 4” (2). Patients diagnosed with GBM currently have a median survival time of less than two years, and their five-year overall survival rate is 5.4% (3). Temozolomide and postoperative radiation are commonly used for the treatment of GBM (4). However, due to its typical late detection, extensive infiltration, and high inter-tumoral as well as intra-tumoral heterogeneity, GBM has limited treatment possibilities (5).

The tumor microenvironment (TME) of GBM is characterized by macrophage infiltration, with GAMs accounting for a significant proportion (30%-50%) of the TME cell, which are mainly replenished by bone marrow derived monocytes (BMDMs) (6, 7). GAMs, which comprise of both microglia present within the brain and BMDMs, display a propensity towards M2 activation in the TME, and exhibit tumor-promoting properties such as stimulating cancer angiogenesis, epithelial-mesenchymal transition, and suppressing immune responses (8). Soon after the tumor cell initiation, both growth and invasion can be promoted and maintained by “educating” GAMs through the release of various tumor-influencing factors, metabolites, extracellular vesicles, as well as direct cell-cell interaction with the malignant cells. At the same time, glioma cells can also produce chemokines and recruit BMDMs to effectively facilitate glioma growth, invasion and migration (9). Therefore, it is essential to thoroughly examine the role of GAMs in GBM progression and identify prognostic markers associated with GAMs, thus opening new avenues for individualized patient treatment.

Recent studies have indicated that scRNA-seq can be used to study intra-tumoral heterogeneity at the cellular level (10). For instance, Darmanis et al. performed scRNA-seq on GBM samples (3589 cells from 4 patients), and results provided valuable insight into the mechanism by which GBM cells infiltrate the surrounding tissues (11). We combined scRNA-seq data with The Cancer Genome Atlas (TCGA) GBM and low-grade glioma (LGG) datasets. After the identification of 14 GAMs-associated prognostic genes, novel prognostic signatures were created. This GAMs-associated characteristic was found to accurately predict the prognosis of GBM patients after validation in the test set.

**Figure 1:**
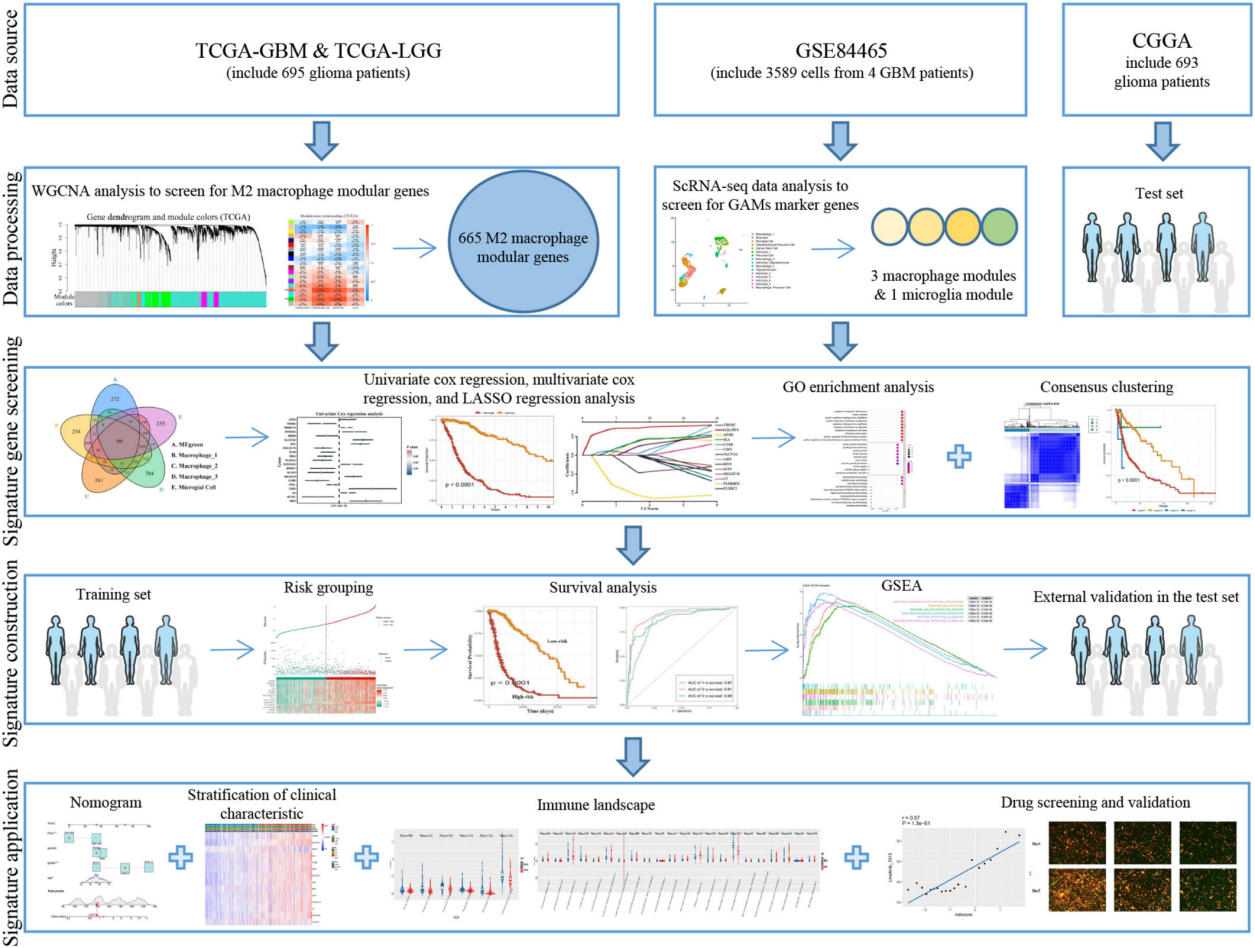
Flowchart.

Histone acetylation plays a crucial role in releasing compacted chromatin to trigger transcription, and any disruption of acetylation homeostasis can affect gene expression (12). Histone deacetylase (HDAC) inhibitors such as Panobinostat, Vorinostat, and Romidepsin have been approved for the treatment of cutaneous T-cell lymphoma and multiple myeloma (12), and several clinical trials are currently underway in GBM (12–14). These drugs can act on the metabolic enhancers at the epigenetic level (15) and can be used in conjunction with DNA-targeting drugs and enzymes due to their ability to regulate chromatin accessibility. We have investigated the impact of Vorinostat on the glioma microenvironment using a prognostic gene risk model. Our findings demonstrate that Vorinostat can display an inhibitory effect on the glioma microenvironment and thus attempted to explain the potential mechanism of action of Vorinostat (**Figure 1**).

## 2 Materials and methods

### 2.1 Sample gathering and processing of genomic data

The TCGA-GBM, TCGA-LGG, GSE84465, and CGGA RNA-seq datasets were acquired from online sources. We utilized the R package “TCGAbiolinks” (16) to retrieve and analyze the TCGA datasets. Information was collected about patient results, primary treatment outcomes, age, sex, and WHO grade.

The GSE84465 single-cell transcriptome profile of GBM, including 3589 cells from 4 GBM patients, was obtained from the GEO database (17). The scRNA-seq data was processed using the “Seurat” package (18), and quality control was performed using the annotation reference uploader. The cell samples were filtered for the mitochondrial gene expression. Cells with >200 genes detected, and genes detected in >3 cells were retained. To identify highly variable genes for downstream analysis, scRNA-seq data was subjected to normalization (LogNormalize) after cell filtering. Subsequently, obvious principal components (PCs) were discovered using principal component analysis (PCA) on genes displaying considerable variability. The uniform manifold approximation and projection for dimension reduction (UMAP) algorithm was applied to the PCs to accomplish cell clustering; the number of PCs choosed 15. The marker genes were found for each cluster of cells using the “FindAllMarkers” function. Finally, the “SingleR” package (19) and the “scCATCH” package (20) were used to annotate the different cell types found in the cell clusters.

### 2.2 Statistical analysis and graph generation

The R language (4.2.1 version) for Windows was employed for statistical analysis and graph generation. The references in other sections of Materials and Methods contain a list of R packages utilized for statistical analysis. Survival was assessed using the Kaplan-Meier method. Unpaired t-test was used to analyze differences in normally distributed data, while the Wilcoxon test was employed for determining the differences in non-normally distributed data. A p-value of 0.05 was used to indicate a significant difference. Multiple survival analysis comparisons use False Discovery Rate (FDR) correction.

### 2.3 Infiltration abundance of M2 macrophages and related survival analyses

We employed four distinct immune infiltration algorithms CIBERSORT, CIBERSORT abs (21), quanTIseq (22), and xCell (23) to calculate the abundance of M2 macrophage infiltration in each TCGA-GBMLGG. A threshold of 50% was utilized in TCGA-GBMLGG to differentiate samples with high and low M2 macrophage content. Survival analysis was performed using the “survival” package (24, pp. 7–37). The Kaplan-Meier survival analysis was used to determine the correlation between glioma survival and the presence of M2 macrophages.

### 2.4 Screening of GAMs related prognostic genes

We analyzed the TCGA-GBMLGG expression data using the “WGCNA” package (25) to identify the genes that were strongly associated to M2 macrophages. This analysis involved grouping glioma patients based on their M2 macrophage content. First, we clustered the samples in the TCGA-GBMLGG dataset. Thereafter a soft-threshold power β was selected based on the lowest power to form a weighted adjacency matrix that conforms to the scale-free topology fit index, which was transformed into a topological overlap matrix. Finally, 18 gene modules were identified by clustering of the average linkage hierarchy with a parameter height of 0.5. To determine the modules that are most relevant to the content of M2 macrophages, a correlation analysis between modules and features was conducted. To obtain genes links to GAMs, the modular genes obtained from the WGCNA package were then intersected with the GAMs markers identified through the scRNA-seq data analysis.

### 2.5 Construction and verification of GAMs-related prognostic characteristics

The genes associated with GAMs that be used to create prognostic features were obtained by univariate Cox regression, multivariate Cox regression and LASSO regression analysis. Based on lambda.min, LASSO regression analysis was performed using the R package “glmnet” (26). Initially, our aim was to demonstrate the connection between the signature genes and prognosis. Based on the following risk scoring model, we conducted a multivariate regression analysis on the training set:


$$riskscore=\sum_{n=1}^{n}\left[coefficient\left(genei\right)*expression\left(genei\right)\right]$$

By employing the formula, patients with glioma were classified into high-risk and low-risk groups based on the median score. Subsequently, a survival analysis was conducted to assess the potential correlation between multivariate cox regression analysis and prognosis. After the glioma samples have been consensus clustered based on the expression levels of characteristic genes, we proceeded to compare the prognoses of these clusters. We utilized the Gene Ontology (GO) database to perform an enrichment analysis of the functions and signaling pathways of the signature genes using the R package “clusterprofiler” (27). According to the results of LASSO regression analysis, the score of each glioma patient in the TCGA-GBMLGG data set was obtained through the following model, and the glioma patients were classified:


$$riskscore=\sum_{n=1}^{n}\left[coefficient\left(genei\right)*expression\left(genei\right)\right]$$

Patients were then divided into low-risk and high-risk categories based on the suitable risk score cut off. The survival rates between the high-risk and low-risk groups were analyzed and compared using Kaplan-Meier survival curves and log-rank testing. We plotted receiver operator characteristic (ROC) curves using the “survivalROC” package (28) to evaluate prognostic model quality. The GSEA software (29) was utilized to analyze significantly enriched pathways, with the KEGG and Reactome databases. The number of permutations was set to 1000. The pathways with a false discovery rate lower than 0.25 and a p-value equal to 0.05 demonstrated significant enrichment. The five most frequent pathways were collected independently in the high-risk group. In addition, the test set was employed to confirm the prognostic characteristics. The “rms” package was used to create a nomogram according to the prognostic and clinical characteristics of the samples. The efficiency of the nomogram was evaluated by employing calibration and ROC curves for the durations of 1, 3, and 5 years.

### 2.6 Analyses of immunity landscape

The “GSVA” package (30) was used to compare immune cell and immune function scores between high-risk and low-risk groups. TIDE (31) predicted immunotherapy response status in high-risk and low-risk categories.

### 2.7 Cell source and handling

Human glioma cell lines A172 and U87MG, human monocytic leukemia cell line THP1, mouse macrophages cell line RAW264.7 were obtained from American Type Culture Collection (ATCC) (Manassas, VA, USA). Mouse glioma cell line GL261 was generously provided by Prof. Tong in Zhejiang University. Human glioma cell line TJ905 was derived from GBM that was resected during surgery and kindly provided by the Laboratory of Neuro-Oncology, Tianjin Neurological Institute. Glioma cell lines A172, U87MG, GL261 and RAW264.7 were cultured in Dulbecco’s Modified Eagle Medium (DMEM) (Gibco, USA) supplemented with 10% Fetal Bovine Serum (FBS) (Procell, China), glioma cell line TJ905 was cultured in DMEM/F-12 (Gibco, USA) supplemented with 10% FBS, and THP1 was cultured in Roswell Park Memorial Institute 1640 Medium (RPMI-1640) (Gibco, USA) supplemented with 10% FBS and incubated in 5% CO2 at 37°C.

### 2.8 Coculture

To initiate conditional co-culture, on the first day, 100 ng/ml phorbol 12-myristate 13-acetate (PMA, Solarbio, China) purchased from MedChemExpress was added to a 24-well culture dish containing THP1 (1×10^5^/well), and at the same time, the culture medium (CM) of U87MG, A172, TJ905 cells was changed to RPMI-1640 until they reached a confluence range of 70%~80%. After 24 hours, RPMI-1640 in glioma cell culture dishes were collected and filtered to process THP1 for 48 hours. RNA was extracted after 48 hours of co-culture for the purpose of performing real-time polymerase chain reaction (RT-PCR). For the Western Blot experiment, similarly, we extracted RNA from RAW264.7 cells treated with GL261 medium for 48 hours, and we extracted protein from RAW264.7 cells after treating them under various conditions for 72 hours. To visualize U87MG and THP1 cell lines, we purchased lentiviruses with fluorescent genes from GeneChem, China. U87MG cells were then transformed into red fluorescent gene (RF), and THP1 was transformed into green fluorescent gene (GF). THP1(GF) (5×10^5^) was then treated with PMA as described above, followed by the addition of U87MG (RF) (5×10^5^) for co-culturing. The fluorescence microscope was utilized to examine the morphology and growth of both cell lines. Vorinostat was used to treat both direct and indirect co-culture models at concentrations of 1.25×10^-3^mM and 2.5×10^-3^mM respectively in fluorescence experiment, and Vorinostat was used at a concentration of 2.5×10^-3^mM in PCR and Western Blot (WB) experiments.

### 2.9 RT-PCR and western blot

Following a previously described method (32), we extracted RNA and performed RT-qPCR. The GoTaq qPCR Master Mix was used to determine the various genes that were expressed (A6001, Promega, USA). The sequences of primers (from Genewiz, China) used for RT-qPCR have been shown in **Supplementary Table S14**. Similarly, we extracted protein and performed WB experiment, the primary antibodies for WB: CD163 (Abcam, ab87099), β-Tubulin (ZSGB-BIO, TA-10).

### 2.10 Drug screening and validation

To identify additional novel potential therapeutic targets and more effective glioma drugs, The Cancer Therapeutics Response Portal (CTRP) and the Genomics of Drug Sensitivity in Cancer (GDSC) database (33) were used to identify the various anticancer drugs whose sensitivity was significantly correlated with the different prognostic genes. Vorinostat was purchased from Beyotime, China.

## 3 Results

### 3.1 Identification of M2 macrophage-associated genes in glioma by WGCNA

**Figure 2:**
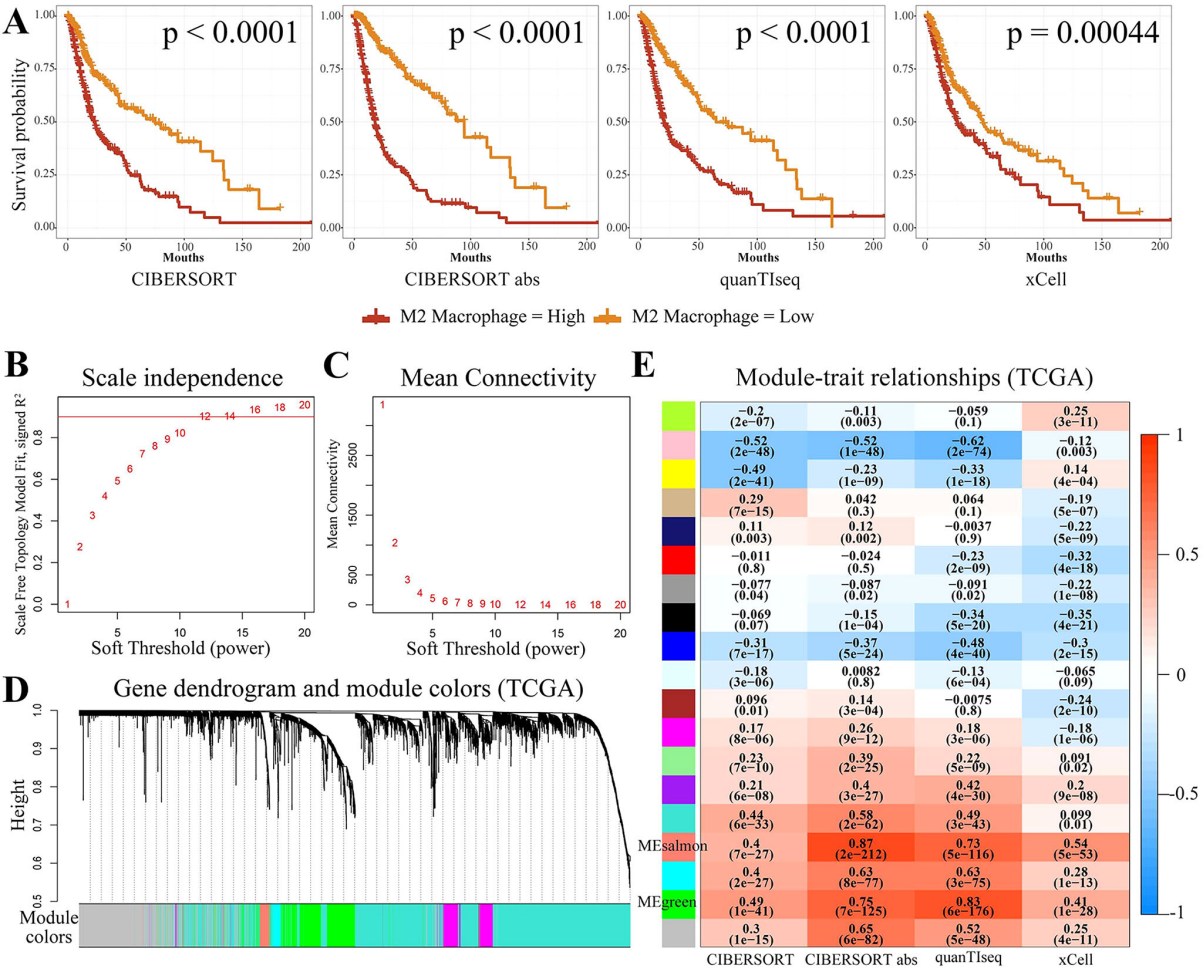
Screening for M2-associated genes in glioma using WGCNA. **(A)** Kaplan-Meier survival curves for the four immune infiltration algorithms, namely, CIBERSORT, CIBERSORT abs, quanTIseq, and xCell, for groups with high and low M2 macrophage concentration. **(B, C)** The WGCNA package’s specifications led to the choice of 12 as the soft threshold power. **(D, E)** There were 18 non-gray modules observed by correlation analysis of the defined modules. MEsalmon and MEgreen were the two modules found to be predominantly associated with M2 macrophages.

Glioma tumor grade was significantly correlated with the abundance of M2-like GAMs (34). To further support the potential association between M2 macrophages and glioma prognosis, the TCGA-GBMLGG data was categorized into high and low M2 macrophage content groups using four different algorithms (CIBERSORT, CIBERSORT abs, quanTIseq, and xCell). This was carried out to further support the association between M2 macrophages and the prognosis of glioma. The results of the Kaplan-Meier analysis demonstrated that the M2 macrophage content was greater in glioma patients with shorter survival time (**Figure 2A**). Therefore, M2 macrophage-associated genes in glioma patients were identified by WGCNA. The first step involved removing specific outlier data from the TCGA-GBMLGG analysis (**Supplementary Figure 1A**), selecting 12 as the optimal soft threshold power (**Figures 2B, C**), and identifying 18 modules using WGCNA (**Figure 2D**). The two modules of MEsalmon (88 genes, **Supplementary Table S1**) and MEgreen (665 genes, **Supplementary Table S2**) exhibited the most significant associations with the high M2 macrophage content (**Figure 2E**). Hence, genes from these two modules were therefore selected for the downstream analysis.

### 3.2 Application of scRNA-seq data to obtain GAMs marker genes of GBM patients

**Figure 3:**
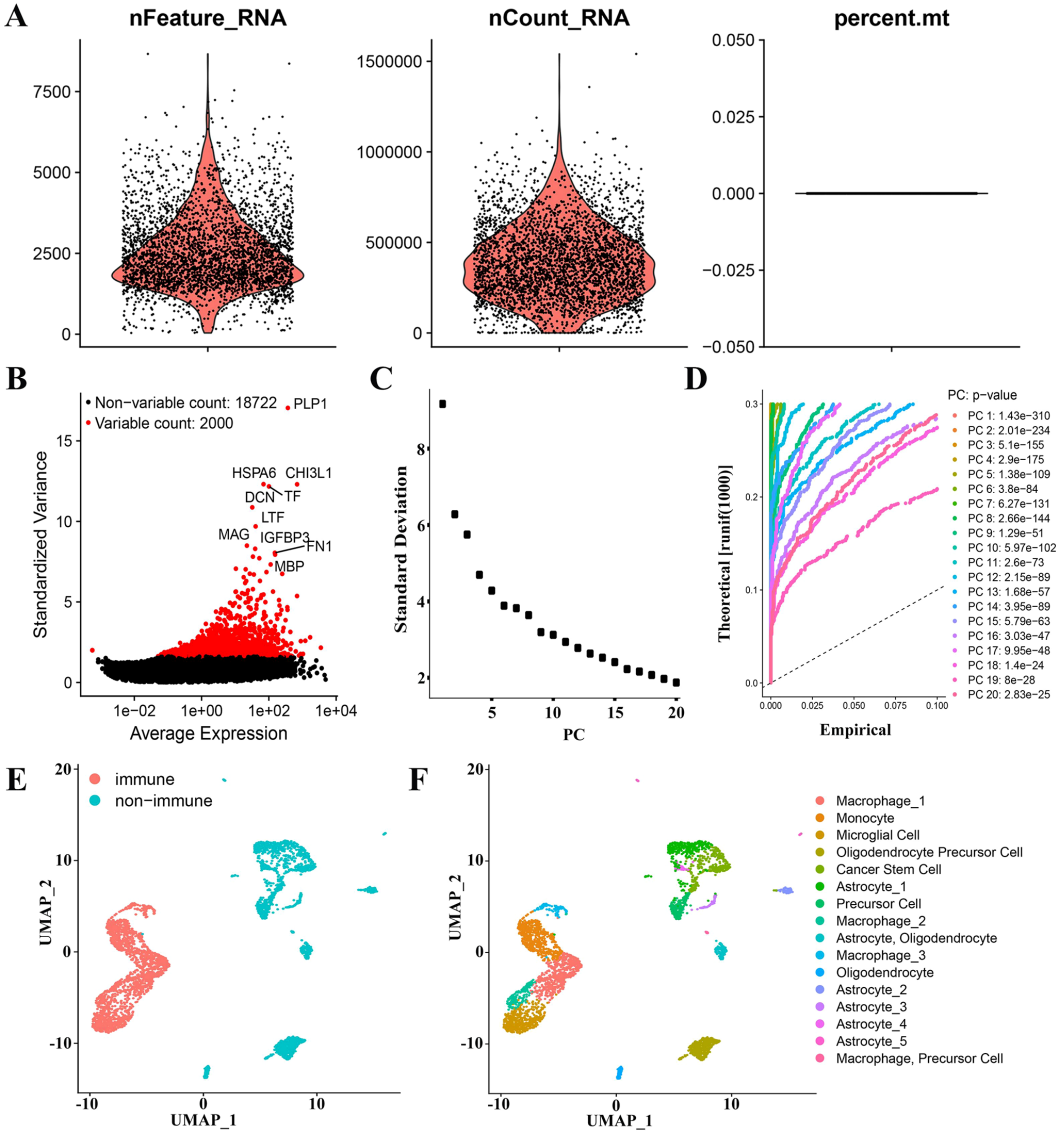
GAMs marker genes of GBM patients were derived through analysis of scRNA-seq data. **(A)** Data quality control of scRNA-seq from GBM cell samples. **(B)** The top 2000 differential genes have been shown in a scatter plot. **(C, D)** The cells were classified using PCA and the top 20 PCs are shown. **(E)** Cells were first classified into “non-immune” and “immune” using the UMAP algorithm. **(F)** Cells were annotated using “singleR” and “scCATCH” R packages.

After quality control, we successfully obtained 20722 genes in 3580 cells. The violin plot (**Figure 3A**) displays the number of genes (nFeature), the number of sequencing counts per cell (nCount), and the proportion of mitochondrial genes (percent.mt). An examination of the correlations revealed a positive correlation between nCount and nFeature (**Supplementary Figure 1B**). The scatter plot of the 2000 variable genes was then generated (**Figure 3B**). The identification of 16 PCs demonstrated the heterogeneity of GBM cells (**Supplementary Figures 1C–E**). UMAP analysis was performed on 16 PCs, and the GBM cells were separated into two distinct groups based on the cell type annotations and UMAP results (**Figures 3B, C**): 1845 immune cells and 1715 non-immune cells (**Figure 3E**). Monocytes, macrophages, and microglia constituted the majority of immune cells. Non-immune cells mainly included glioma stem cells, astrocytes, and oligodendrocytes (**Figure 3F**). Three sets of marker genes for macrophages (**Supplementary Tables S3**-**S5**) and one set specific for microglia (**Supplementary Table S6**) were removed for the downstream analysis.

### 3.3 Screening of GAMs related prognostic genes

**Figure 4:**
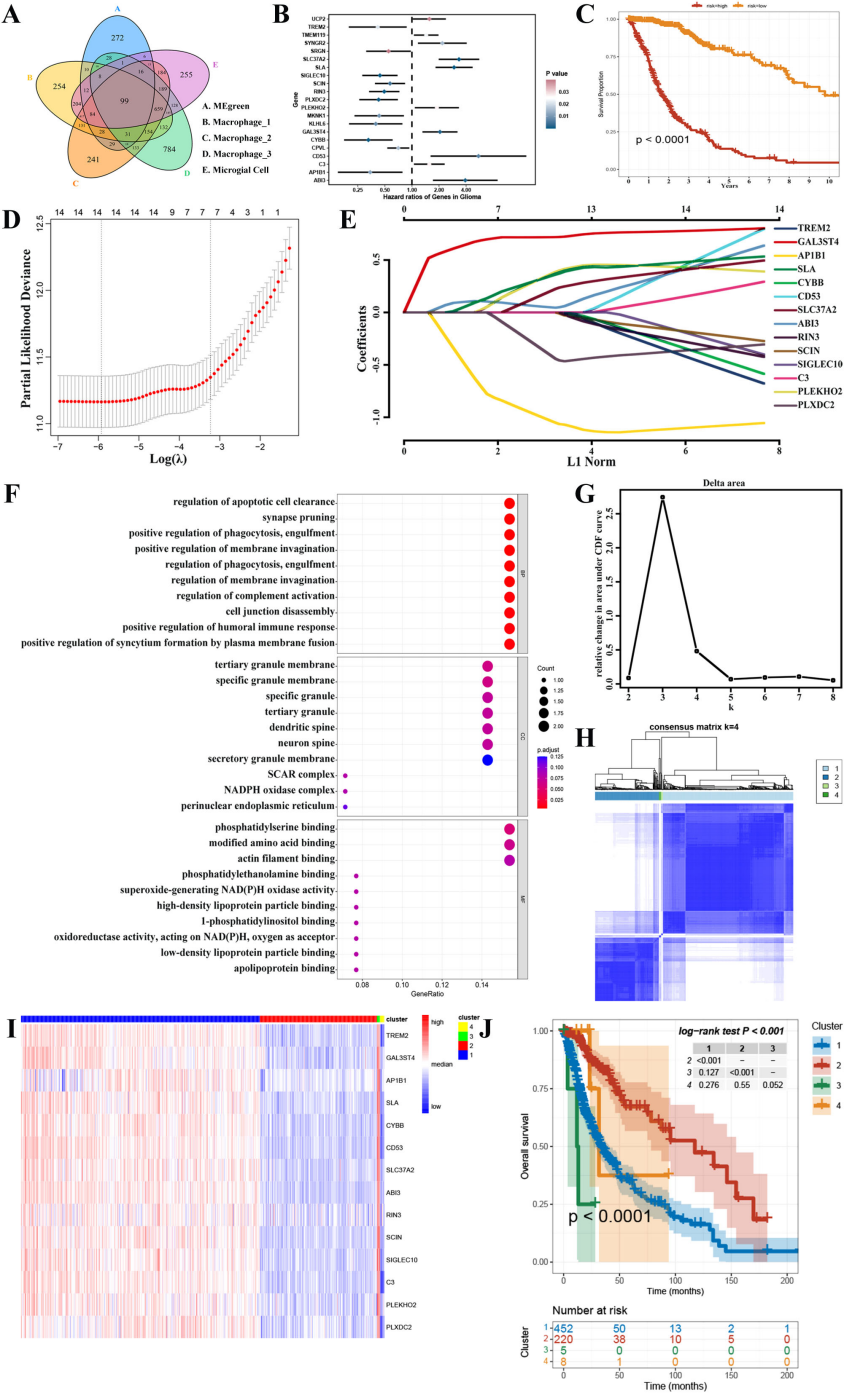
Screening for GAMs-associated prognostic genes. **(A)** The candidate genes associated with GAMs. **(B)** The results of a univariate cox regression study identified 21 distinct genes associated with GAMs. **(C)** Multivariate cox regression analysis was used to construct a survival analysis for the risk stratification. **(D, E)** Signature genes were discovered using LASSO regression analysis. **(F)** GO database functional enrichment analysis. **(G, H)** Consensus clustering revealed that K=4 was the best, and TCGA-GBMLGG was divided into 4 clusters. **(I)** The heatmap depicted the variations in gene expression between the 4 clusters. **(J)** The Kaplan-Meier survival curve showed the discrepancies in the survival rates among the four clusters.

After analyzing the intersection between the four gene sets obtained from scRNA-seq and the two gene modules obtained from WGCNA, it was discovered that only the MEgreen module contained potential genes related to GAMs. The module yielded a total of 99 candidate genes (**Figure 4A**, **Supplementary Figure 2A**). Initially, through univariate cox regression analysis, it was identified that 21 genes associated with GAMs were found to be related with the prognosis of GBM (**Figure 4B**, **Supplementary Table S7**). Next, by multivariate cox regression analysis, 16 GAMs genes (**Supplementary Table S8**) were discovered to be linked to the prognosis of GBM patients (**Figure 4C**).

In addition, 14 prognostic signature genes were identified through the final LASSO regression analysis after eliminating the two genes with p-values less than 0.05 in the multifactorial cox regression analysis (**Figures 4D, E**). These included *Triggering Receptor Expressed On Myeloid Cells 2* (*TREM2*)*, Galactose-3-O-Sulfotransferase 4* (*GAL3ST4*)*, Adaptor Related Protein Complex 1 Subunit Beta 1* (*AP1B1*)*, Src Like Adaptor* (*SLA*)*, Cytochrome B-245 Beta Chain* (*CYBB*)*, CD53, Solute Carrier Family 37 Member 2* (*SLC37A2*)*, ABI Family Member 3* (*ABI3*)*, Ras And Rab Interactor 3* (*RIN3*)*, Scinderin* (*SCIN*)*, Sialic Acid Binding Ig Like Lectin 10* (*SIGLEC10*)*, Complement C3* (*C3*)*, Pleckstrin Homology Domain Containing O2* (*PLEKHO2*) *and Plexin Domain Containing 2* (*PLXDC2*). The expression of these 14 genes in the GBM microenvironment is depicted in **Supplementary Figure 3**. In the GO database, a total of 822 terms were identified as having a significant enrichment of prognostic genes; the bubble graph displays the top 10 of these terms (**Figure 4F**). In addition, the expression of prognostic genes was used to cluster the TCGA-GBMLGG samples into several groups. Consensus clustering demonstrated improved outcomes when K=4 (**Figures 4G, H**). Thus, 4 clusters of glioma patients were generated. Heatmaps were then employed to show the differences in gene expression between these 4 groups (**Figure 4I**, **Supplementary Figure 7**). In addition, there was a clear difference observed in survival between the 4 groups (**Figure 4J**). The above multivariate cox regression and consensus clustering indicated that these 14 genes possess significant prognostic value in glioma patients.

### 3.4 Construction of GAMs-related prognostic features and external validation

**Figure 5:**
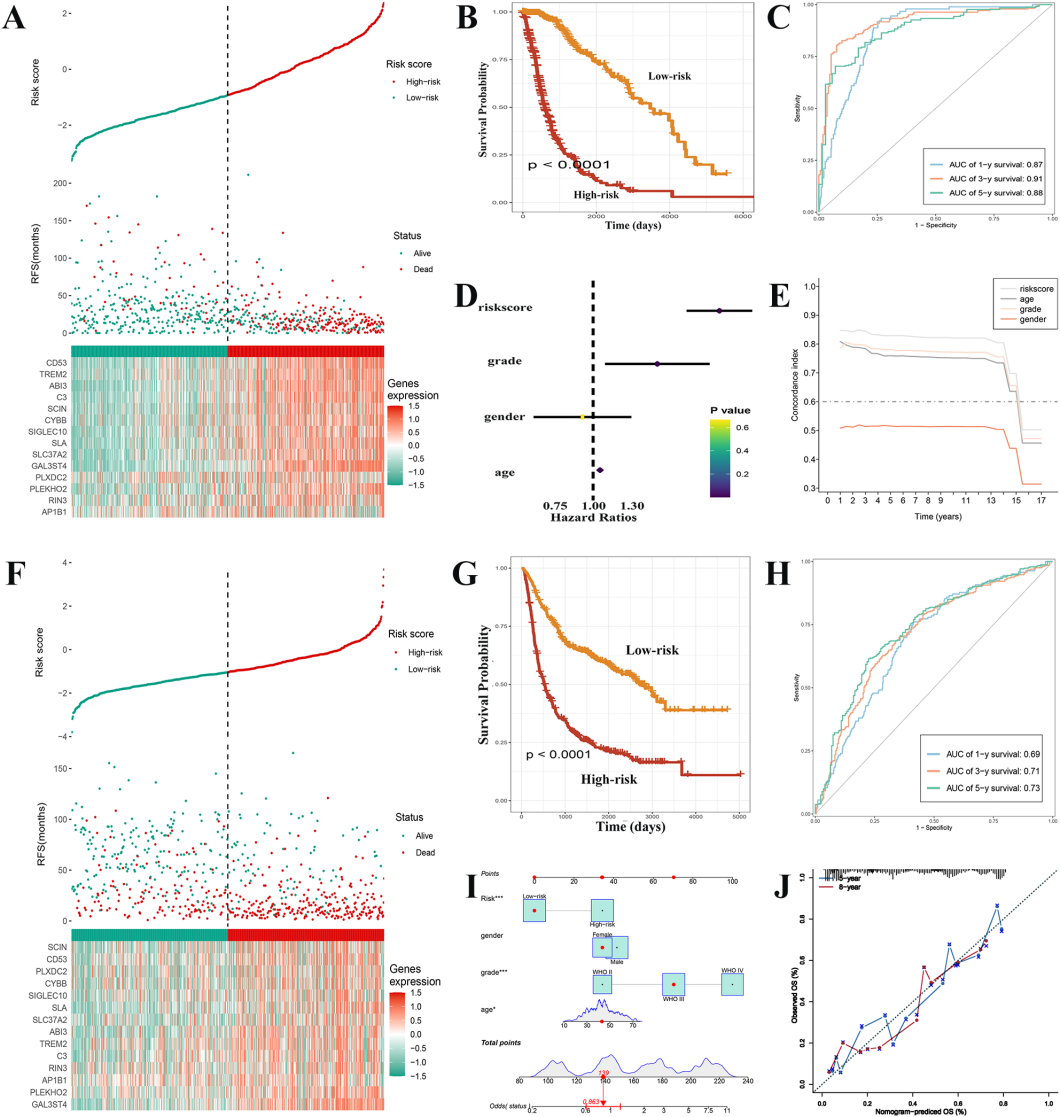
Construction of GAMs-related prognostic features and external validation. **(A)** Comparison of the survival status of glioma patients with different risk scores in the training set. **(B)** The high-risk group of training set exhibited a much poorer prognosis, according to Kaplan-Meier survival curves. **(C)** ROC curves with their AUCs for 1, 3, and 5 years, respectively. **(D, E)** In contrast to other indicators, the risk score was discovered to serve as an independent risk factor for the survival status using univariate analysis and the C-index. **(F)** Patients with gliomas in the high-risk and low-risk categories of the test set were compared for the survival status and risk score. **(G)** The high-risk group in the text set had a lower survival time, as indicated by the Kaplan-Meier survival curve. **(H)** ROC curves with their AUCs for 1, 3, and 5 years, respectively. **(I)** Nomogram based on the various clinical markers and risk scores. The results of a calibration curve **(J)** demonstrated the consistent reliable performance of the nomogram.

The estimated risk score for each sample in the TCGA-GBMLGG dataset was determined using the results from the LASSO regression analysis (**Supplementary Table S9**) and the expression levels of genes associated with prognosis. Thereafter, according to the corresponding risk score cut-off point (-0.92), the training set was divided into two distinct groups (**Figure 5A**). Interestingly, patients in the low-risk group had an overall better prognosis, surpassing those in the high-risk group in terms of overall survival (OS) (**Figure 5B**). When evaluating the efficiency of the risk model, the effectiveness was measured using ROC curves. The areas under the curves (AUC) at 1, 3, and 5 years were found to be 0.87, 0.91, and 0.88 respectively (**Figure 5C**). The risk score was found to be a more reliable prognostic predictor in comparison to other factors and acted as an independent factor influencing survival, according to the findings of univariate analysis (**Figure 5D**) and concordance index (C index) (**Figure 5E**). Based on this risk score, we performed GSEA functional enrichment analysis. The high-risk category was observed to be enriched in different pathways such as DNA replication, cell cycle, and especially integrins and interleukins (**Supplementary Figures 2B, C**, **Supplementary Tables S10**, **S11**). The enrichment of integrins and interleukins pathways indicates the recruitment of macrophages by tumor cells and the M2 polarization of macrophages in gliomas, which is also a long-term focus of our team. The enrichment of pathways related to cell cycle, DNA replication, and other processes is associated with the malignancy of tumors. These enriched pathways provide part of the mechanism and basis for the short lifespan of high-risk patients. We then further verified the prognostic function in the test set to confirm its reliability. The samples, similar to the training set, the samples were categorized (**Figure 5F**). It was noted that the prognosis of patients in the high-risk group was worse than that of patients in the low-risk group (**Figure 5G**), with AUCs at 1, 3, and 5 years in the test set of 0.69, 0.71, and 0.73, respectively (**Figure 5H**). These findings validated the usefulness of prognostic factors linked to GAMs in predicting prognosis of glioma patient. In order to enhance the accuracy of patient survival prediction, we produced a nomogram (**Figure 5I**) according to our predictive risk model and other clinicopathological patient indications. In addition, calibration curve and ROC curve results of nomogram displayed its dependability (**Figure 5J**).

### 3.5 Clinicopathologic features are analyzed based on the prognostic signature

**Figure 6:**
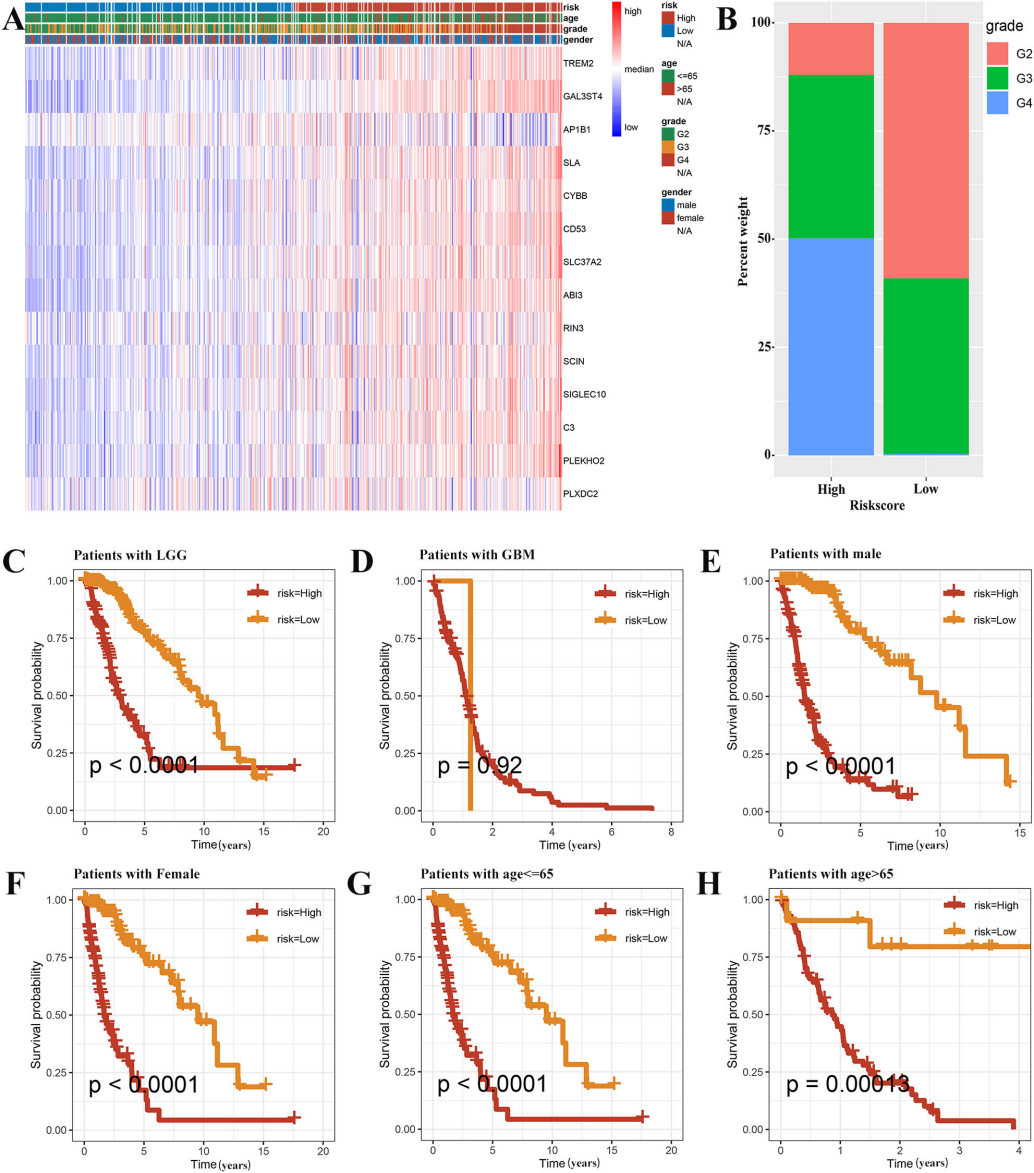
Prognostic analysis based on the clinicopathological categorization. **(A)** A heat map was employed to depict the variations in gene expression and clinicopathologic characteristics among the two risk groups. **(B)** The histogram revealed risk scores correlated with the various clinicopathological characteristics. The results of prognostic analysis stratified by the tumor grade **(C, D)**, sex **(E, F)**, and age **(G, H)** were presented using Kaplan-Meier survival analysis.

The survival rates significantly varied between the high-risk and low-risk groups, with significant differences in their clinicopathological characteristics. A heat map of the clinicopathological signature and the expression of genes associated with the signature in two different risk groups has been shown in **Figure 6A**. The proportion of WHO grade 4 patients in the high-risk group was found to be significantly higher, thus indicating a statistically significant difference in tumor grade between the two groups. (**Figure 6B**). Almost all GBM patients were high-risk, and in LGG patients, the prognosis of high-risk group was worse (**Figures 6C, D**). In addition, when patients were grouped according to age and gender, the survival rate of the high-risk group was significantly lower. (**Figures 6E-H**).

### 3.6 Immune cells, immune function, and the landscape of immunotherapy in relation to risk signatures

**Figure 7:**
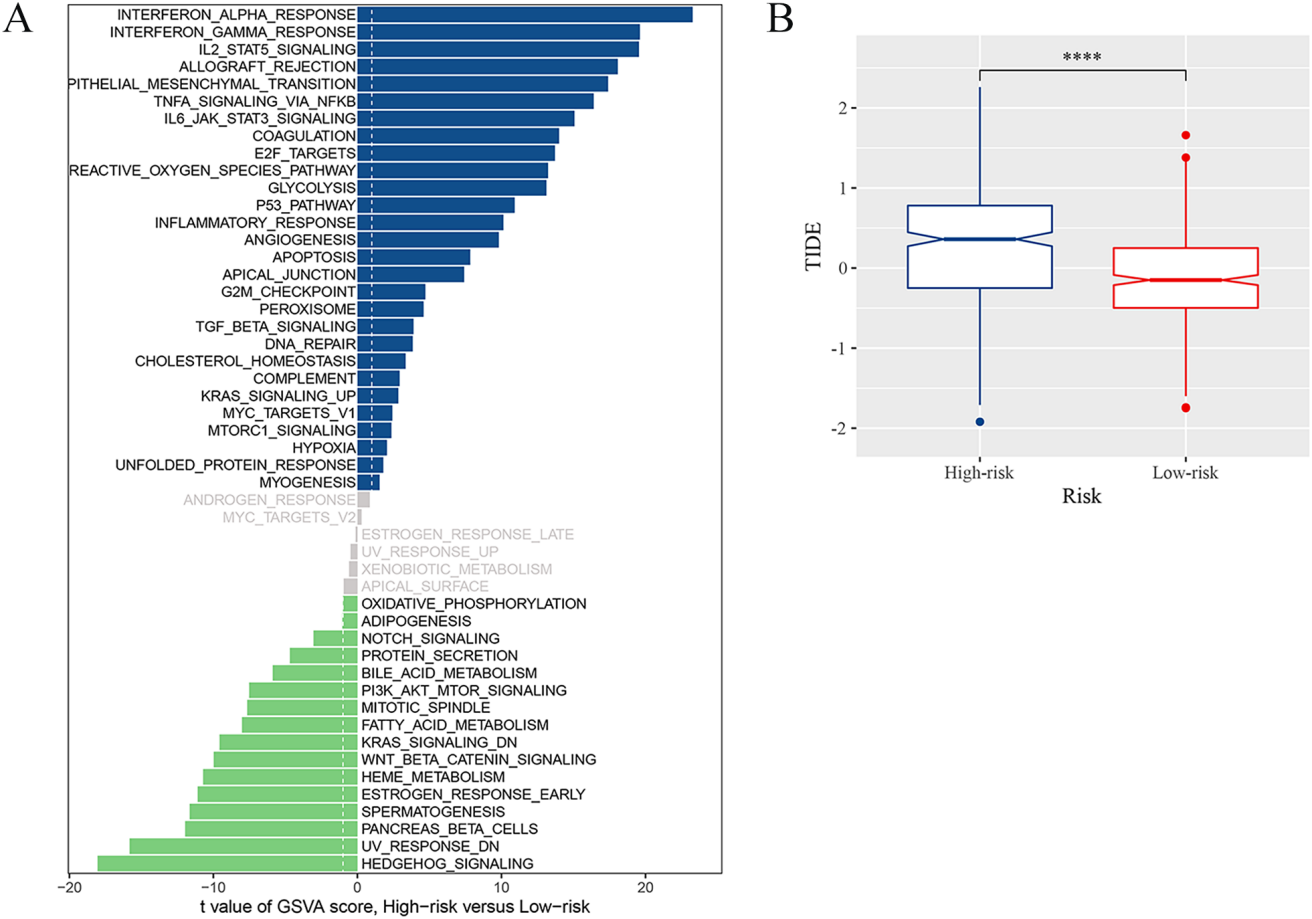
Immune landscape associated with the risk signatures. **(A)** Analysis of GSVA among the patients of two risk groups. **(B)** TIDE score for high-risk and low-risk groups (****p <0.0001).

We assessed the immune microenvironment of glioma by utilizing four immune infiltration algorithms to determine the abundance of M2 macrophages, based on the risk grouping. Interestingly, it was found that the high-risk group had a larger concentration of M2 macrophages and CD4+ effector T cells compared to the low-risk group as identified by all immune infiltration algorithms (**Supplementary Figure 4**). By gene set variation analysis (GSVA), our results were enriched in “TNFα signaling via NF-κB” and “Interferon gamma response”, which were confirmed as TME local and global tissue modifiers (35), indicating the complexity of the immune microenvironment in the high-risk group. Moreover, we found that pathways such as “epithelial mesenchymal transition”, “IL6-JAK-STAT3 signaling”, and “P53 pathway”, which are highly associated with tumor malignancy progression, were enriched in the high-risk group, suggesting a close link between the high abundance of GAMs and tumor malignancy progression in the high-risk group. Regrading immunotherapy, the TIDE score was significantly elevated in the high-risk group was higher (**Figure 7B**), thus suggesting that patients in the high-risk group had a high possibility of immune escape and a reduced efficacy of immunotherapy. Numerous immunological checkpoint-related genes (36) were found to show differential expression between the two groups (**Supplementary Figure 5**). These included *BTN3A1*, *CD276*, and *CD274*, which exhibited higher expression in the high-risk group.

### 3.7 Prediction and verification of potential anticancer drugs

**Figure 8:**
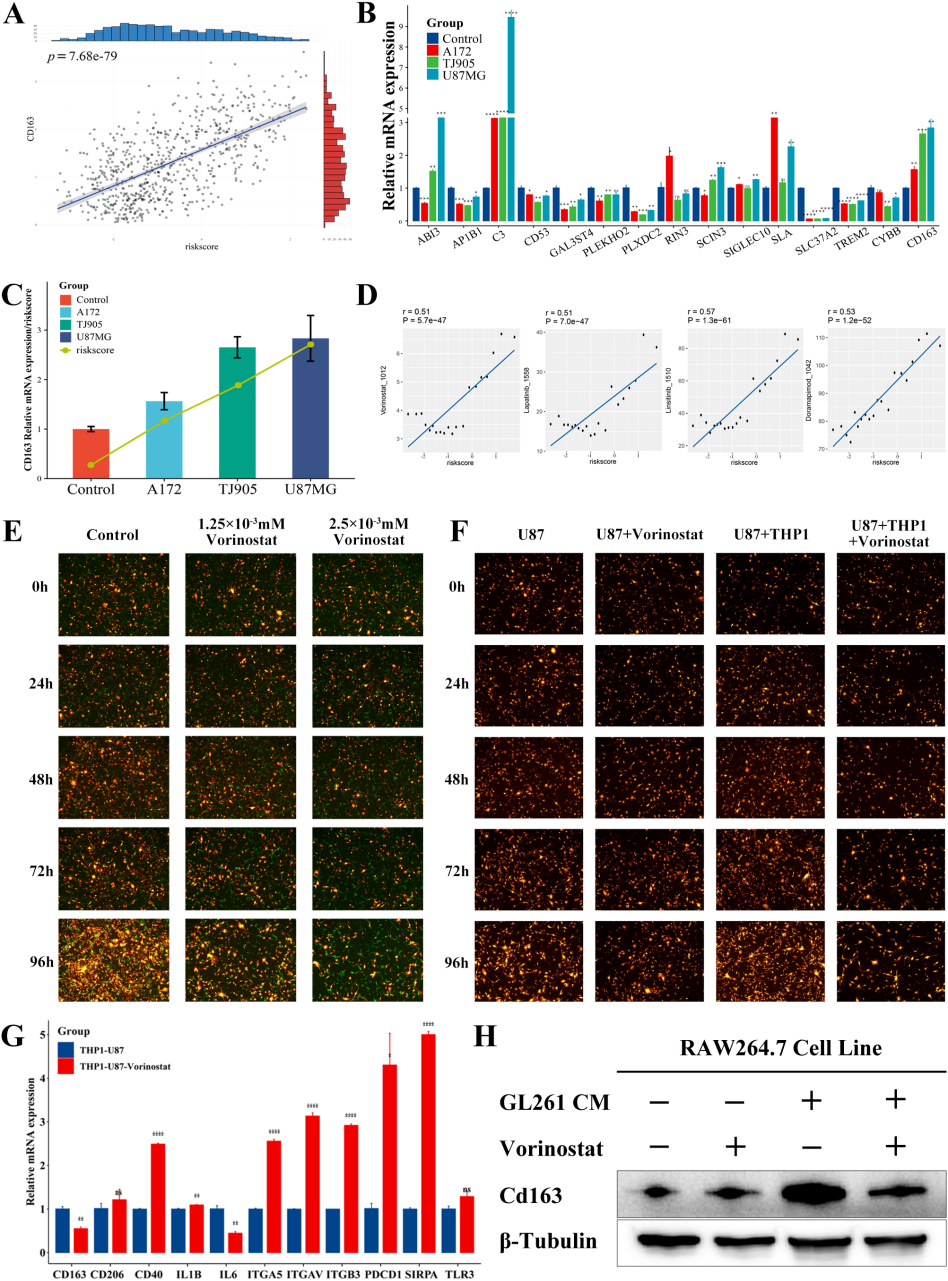
Prediction and validation of the potential anticancer drugs. **(A)** Correlation between CD163 expression and risk score in the TCGA database (p <0.0001). **(B)** Differences in mRNA expression of various prognostic genes and CD163 in the indirect co-culture model (*p <0.05; * *p <0.01; * * *p <0.001; * * * *p <0.0001). **(C)** Expression of CD163 and risk score in the indirect co-culture model. **(D)** Potential anticancer drugs were predicted using signature genes and a risk score. **(E)** The U87MG (RP) was cocultured with the PMA-induced adherent THP1 (GP), and the treatment groups were then treated with Vorinostat. **(F)** Analysis of growth of U87MG and U87MG cocultured with PMA-induced adherent THP1 before and after Vorinostat treatment. **(G)** Expression of macrophage-related immune checkpoints of THP1 before and after the treatment with Vorinostat in the U87MG-THP1 indirect co-culture model (*p <0.05; * *p <0.01; * * *p <0.001; * * * *p <0.0001). **(H)** The expression of Cd163 in RAW264.7 cells at the protein level, with GL261 conditioned medium and Vorinostat as variables.

The TCGA database revealed a link between the risk score and the M2 marker CD163 (**Figure 8A**). First, we indirectly co-cultured THP1 with the culture media of tumor cells to study the expression of prognostic genes in glioma-associated macrophages, in order to further explore the therapeutic applicability of the various prognostic genes. When the tumor culture media of A172, TJ905 and U87MG cells were co-cultured with THP1 after PMA treatment, it was observed that the expression of *C3* and *SLA* genes was considerably elevated, while the expression of *AP1B1*, *CD53*, *GAL3ST4*, *PLEKHO2*, *PLXDC2*, *SLC37A2*, and *TREM2* genes showed significant reduction (**Figure 8B**). The risk model formula was used to calculate the scores of the four treatment groups. The results showed a correlation between the risk scores and the CD163 expression (**Figure 8C**).

Based on the expression pattern of the risk model, we investigated the potential associated between GAMs markers genes and drug sensitivity. We utilized the CTRP and GDSC datasets to gain insights into the clinical application of prognostic genes (**Supplementary Table S12**). There were four drugs identified with a correlation greater than 0.5 with the risk score (**Figure 8**): Linsitinib (correlation=0.57, P<0.001), Doramapimod (correlation=0.53, P<0.001), Vorinostat (correlation=0.51, P<0.001), Lapatinib (correlation=0.51, P<0.001). Among them, Vorinostat exhibited the lowest IC50. Moreover, in the direct co-culture model, Vorinostat demonstrated a significant inhibitory effect on the U87-THP1 co-culture model (**Figures 8E, F**, **Supplementary Figure 6**), indicating that in can affect the reciprocal tumorigenicity between the tumor cells and macrophages, with therapeutic implications for this prognostic model. We used a mouse co-culture model to analyze the expression of 14 prognostic genes after co-culture and Vorinostat treatment, and found that genes such as Abi3, Cd53, Cybb, Rin3, Sla, Sla37a2, and Cd163 showed an upregulation after co-culture and a downregulation after Vorinostat treatment (**Supplementary Figure 8**), suggesting that Vorinostat might affect the expression of Cd163 and the malignancy of GAMs by modulating these genes. As shown in **Figure 8F** and **Supplementary Table S13**, Vorinostat primarily inhibited glioma cell growth by affecting macrophages. We further investigated the effect of Vorinostat on macrophage-related immune checkpoints of GAMs using an indirect co-culture model. Interestingly, Vorinostat significantly reversed the expression of CD163 and Cd163 in GAMs (**Figures 8G, H**), which could be related to the downregulation of IL-6-related signaling pathways. However, unexpectedly, the levels of PD1, SIRPα, and certain integrins were increased, implying that the utilization of Vorinostat along with PD1 or other targeted drugs may be beneficial in the treatment of glioma.

## 4 Discussion

Glioma is a complex tumor composed of both tumor and non-tumor cells. It has been established that most of these non-tumor cells are GAMs, including intracranial resident microglia and myeloid-derived macrophages, which play critical role in glioma development, progression, and response to therapy (8). Microglia are intracranial resident macrophages that originate from the yolk sac and persist by replenishing themselves. They play important roles during each specific developmental stage of the central nervous system (37). BMDMs are recruited early in glioma genesis and localize in perivascular niches, thereby potentially contributing to further glioma progression (6). As GAMs infiltrate brain tumors, microglia exhibit distinct transcriptional profiles and activation states in the co-educational environment of tumors due to their distinct chromatin landscapes derived from BMDMs (38). Therefore, understanding the spatiotemporal characteristics of each component of GAMs is crucial for effectively treating glioma.

Multiple pathways have been demonstrated to contribute to the promotion of glioma development when glioma cells interact with GAMs. For example, the increase in microglia is associated with reduced expression of the chemokine receptor CX3CR1, which is responsible for guiding macrophage migration (39), while the activation of microglia is linked with increased c-Jun-NH2-kinase signaling (40). Microglia secrete various paracrine factors, including hyaluronidase, to promote proliferation of astrocytes with Neurofibromatosis type 1 gene (*NF1*) heterozygosity (41). Osteopontin (OPN) plays an important role in the immunosuppressive properties of macrophages, acting as a potential chemokine that facilitates infiltration of macrophages into GBM by interacting with integrin α_v_β_5_ (42). Glioma stem cells (GSCs) secrete periostin (POSTN), a protein primarily found in the perivascular zone, and recruit GAMs through integrin α_v_β_3_ to promote tumor progression (34). GAMs can also induce Matrix metalloproteinase-9 (MMP-9) expression and increase the invasiveness of GSCs by releasing Transforming Growth Factor-β (TGF-β) (43).

The growing interest in GAMs-based therapies stems from the crucial role GAMs play in the development of glioma. In terms of immune checkpoints, CD47 is a cell surface molecule which functions as an immune checkpoint, while signal regulatory protein α (SIRPα) is a heterogeneous receptor found on macrophages. CD47 enhances the ability of tumor cells to evade macrophage phagocytosis by participating in SIRPα (44). In the GBM TME, the inhibition of CD47 can effectively re-educate GAMs to unlock the therapeutic potential of tumor cell phagocytosis (45, 46). In preclinical studies, GSC-specific chimeric antigen receptor (CAR) macrophage/microglia based on the nanotransporter-hydrogel superstructure showed demonstrated significant tumoricidal immunity and the ability to inhibit GBM recurrence in preclinical studies (47). As a small molecule inhibitor, RRx-001 can polarize the low phagocytic M2 phenotype of tumor-associated macrophages to a high phagocytic M1 phenotype, and can decrease the levels of CD47 on cancer cells and SIRPα on macrophages simultaneously (48). It showed low toxicity and antitumor activity in the preclinical studies.

We evaluated the M2 macrophage content of TCGA-GBMLGG patients using four different immune infiltration scores and observed that glioma patients with a higher M2 macrophage content had significantly worse prognosis. The correlation between M2 macrophages and glioma prognosis was confirmed. We extracted M2 macrophage module genes by WGCNA, intersected with 3 groups of macrophages and 1 group of microglia identified by the single-cell database, and ultimately 99 M2-like GAMs-associated genes were obtained. After univariate regression analysis, multivariate regression analysis and LASSO regression analysis, 14 different prognosis-related genes (*TREM2*, *GAL3ST4*, *AP1B1*, *SLA*, *CYBB*, *CD53*, *SLC37A2*, *ABI3*, *RIN3*, *SCIN*, *SIGLEC10*, *C3*, *PLEKHO2* and *PLXDC2*) were screened to construct the prognostic features.

According to literature findings, *TREM2* has previously been found to play an important role in the function of microglia in neurodegenerative diseases. With the development of single-cell sequencing, it has also been found to be highly expressed in myeloid subpopulations of GBM patients and is associated with poor prognosis. Knockdown of *TREM2* can reverse the M2-like polarization of GAMs and may even facilitate the application of immune checkpoint therapies in GBM (49, 50). *SLA* is found in the amplified region in GBM, thereby suggesting that its overexpression may be important in the development of GBM (51). The role of *CYBB* in GBM radioresistance and its potential as a prognostic marker for radiotherapy in GBM patients have been identified (52). In a study comparing the effects of GBM regorafenib and lomustine treatment, it was found that regorafenib group with higher *CD53* expression had a longer survival rate than the lomustine group (53). *CD53* is a member of the tetraspanin family, known for its involvement in various signal transduction processes and influence on cell development, activation, growth, and mobility. *CD53* has been identified as a tumor initiation marker in cancer stem cells. *ABI3* has also been reported as a key gene of disulfidptosis in low-grade gliomas, which is associated with patient prognosis and immune microenvironment (54). *SCIN* expression is associated with MMP2/9 activation, immune infiltration, and a decreased survival rate. It can serve as a potential target gene for glioma immunotherapy and a biomarker for predicting the survival (55). *SIGLEC10* play a significant role in the regulation of inflammation in glioma through its close association with mediators, cells, and pathways involved in this process. In the TME, *SIGLEC10* can act in conjunction with some immune checkpoints to promote the progression and metastasis of glioma by affecting the immune response (56). *C3* is a critical component of the innate immune system and is involved in the regulation of the epithelial-mesenchymal transition, local immune response, and the TME, It can be used as a diagnostic biomarker and a potential target for precision therapy in LGG patients (57). The current focus of research on *C3* in glioma is primarily centered on the tumor itself, with limited investigation into its presence and role in the surrounding glioma microenvironment (58, 59). Current advances in technologies such as single-cell sequencing, are expected to provide novel insights and methodologies for studying the genes associated with M2-like GAMs.

After four different groups were formed using unsupervised consensus clustering of the TCGA-GBMLGG data based on the 14 genes associated with prognosis and revealing a potential relationship between these 14 genes and prognosis, Kaplan-Meier survival analysis exhibited significant differences among these four groups. The patients’ risk score was calculated based on our prognostic criteria, and the patients were thereafter divided into high-risk and low-risk groups using the optimal cut-off value. We found that the prognosis of patients with high-risk patients was significantly worse than that of low-risk patients. Through GSEA enrichment analysis, we observed that the high-risk group was enriched in M2 macrophages and cancer-related pathways, which implies that investigating the significance of integrins (60, 61) and interleukins in the context of glioma warrants further exploration.

The above results demonstrate that the prognostic features have high performance and can effectively predict glioma prognosis independently of other factors in the training set. Additionally, we conducted an external validation of the CGGA database and demonstrated the wide applicability and reliability of its prognostic features in the test set. We created nomograms according to our feature-based risk scores and the patients’ clinicopathologic indicators to generate different metrics that can be used to assess patients’ prognosis from different perspectives.

Our prognostic signature not only accurately predicted the prognosis of glioma patients, but it also highlighted the possible relationship between risk groups, the immune system, and response to immunotherapy. Regarding immune cells, we used four immune infiltration scores to evaluate the content of immune cells. The high-risk group exhibited a significantly greater number of M2 macrophages in comparison to the low-risk group. However, the abundance of other cells in the glioma microenvironment was not as high and the findings varied across the algorithms. In terms of immune function, “interferon α response”, “interferon γ response”, and “IL-2 STAT5 signaling pathway” were enriched in the high-risk group. Based on our risk grouped model, the heterogeneity of the glioma TME could be the underlying factor for variations in glioma prognosis. It is imperative to extensively assess the applicability of each immune infiltration algorithm within the glioma microenvironment, and to employ both immune function and single-cell sequencing analysis to gain a more comprehensive understanding of the glioma immune landscape.

We next studied and evaluated potential cancer drugs together with signature genes and risk groups. We identified Vorinostat as the drug most sensitive to the risk score. It has been reported that Vorinostat can enhance the level of histone acetylation by inhibiting the activity of histone deacetylase. It can inhibit the growth of GBM cells under *in vivo* and *in vitro* settings (62), and improve the prognosis of GBM patients in combination with radiotherapy (63). However, the relationship between drugs and characteristic genes needs to be further investigated. The co-culture model demonstrated that Vorinostat can affect glioma cell growth through its action on macrophages. Thus, to investigate the mechanism of action of Vorinostat, we examined various macrophage-related immune checkpoints. Polarized M2 macrophages can promote tumor phenotypes, including glycolysis and proliferation, through the release of IL6 (64). The decrease in CD163 levels in THP1 cells in the indirect co-culture model following Vorinostat treatment could potentially be associated with the reduction in IL6 expression. Interestingly, the treatment of Vorinostat upregulated the expression of CD40, PD1, SIRPα and some integrins. The epigenetic regulation of Vorinostat is expected to produce identifiable changes in immunity and metabolism, as it plays a crucial role in maintaining a balance between histone acetylation and deacetylation (13–15, 65). Our research has discovered the ability of Vorinostat to reverse the tumor immune microenvironment, so the combination of HDAC inhibitors and targeted drugs could be a promising approach for targeting the immune microenvironment of glioma. We will continue to focus on the role of Vorinostat and other histone deacetylase inhibitors in the glioma-associated immune microenvironment.

## Data Availability

The data presented in the study are deposited in the **Supplementary Material**. The raw bulk RNA-seq data are sourced from the TCGA database (Project ID: TCGA-GBM, TCGA-LGG) and the CGGA database (Dataset ID: mRNAseq_693). The raw single-cell RNA-seq data are sourced from the GEO database (Accession Number: GSE84465).
