# Supplementary material for: Interrogation of macrophage-related prognostic signatures reveals a potential immune-mediated therapy strategy by histone deacetylase inhibition in glioma
Source: Front Oncol. 2025 Jun 6;15:1554845. doi: 10.3389/fonc.2025.1554845 (PMC12179195; doi:10.3389/fonc.2025.1554845)
Supplement: Supplementary file 14 [file Table1.docx]

| gene | primer sequences |
| --- | --- |
| ABI3 | FP 5'-ATCGCCCCAGAGAACCTACC-3', RP 5'-GCTCTTTCGAGACAGGGTGC-3'. |
| ACTB | FP 5'-CATGTACGTTGCTATCCAGGC-3', RP 5'-CTCCTTAATGTCACGCACGAT-3'. |
| AP1B1 | FP 5'-GACCTCATCTCCGACTCTAACC-3',  RP 5'-GTGAGACTCGGCAATTTCTGA-3'. |
| C3 | FP 5'-GGGGAGTCCCATGTACTCTATC-3',  RP 5'-GGAAGTCGTGGACAGTAACAG-3'. |
| CD53 | FP 5'-TCAACTTGCTCTTTTGGATCTGT-3',  RP 5'-AGAACGACATAAGCAGACACTTG-3'. |
| CYBB | FP 5'-ACCGGGTTTATGATATTCCACCT-3',  RP 5'-GATTTCGACAGACTGGCAAGA-3'. |
| GAL3ST4 | FP 5'-CCTGAAGACACATAAATCCGGG-3',  RP 5'-GCCTGGAAGAGCTTTGGGTA-3'. |
| GAPDH | FP 5'-GTGGTCTCCTCTGACTTCAACA-3',  RP 5'-TTGCTGTAGCCAAATTCGTTGT-3'. |
| PLEKHO2 | FP 5'-CGAAAACACCGCTTTATCCTGC-3',  RP 5'-TTGCCTCGGTTAATCCCTTCA-3'. |
| PLXDC2 | FP 5'-CCAGTTTCAGTTCGCCGATG-3',  RP 5'-TGTCTACCGCCTTGAGAAAGT-3'. |
| RIN3 | FP 5'-TTTCCTTCTCTGAACGAAAGCTC-3',  RP 5'-ACACAGTAGAACGCAATCAATCT-3'. |
| SCIN | FP 5'-ATGGCTTCGGGAAAGTTTATGT-3',  RP 5'-CATCCACCATATTGTGCTGGG-3'. |
| SIGLEC10 | FP 5'-AAGGGACTCATCTCAACGGC-3',  RP 5'-CCGTCTCTTCGGTAGAATCTTCA-3'. |
| SLA | FP 5'-CGACTTCCTTGCCGTGCTAA-3',  RP 5'-TCTCGACCAGTGCTAAGAGAA-3'. |
| SLC37A2 | FP 5'-CCGGGAGTCTGGTTCTTCC-3',  RP 5'-ACGATACTGATAGGCTTCCTGG-3'. |
| TREM2 | FP 5'-CATCACAGACGATACCCTGGG-3',  RP 5'-GCAGATGGGAGCCTTGAGAT-3'. |
| SIRP A | FP 5'-GGCCTCAACCGTTACAGAGAA-3',  RP 5'-GTTCCGTTCATTAGATCCAGTGT-3'. |
| TLR3 | FP 5'-TTGCCTTGTATCTACTTTTGGGG-3',  RP 5'-TCAACACTGTTATGTTTGTGGGT-3'. |
| IL1B | FP 5'-ATGATGGCTTATTACAGTGGCAA-3',  RP 5'-GTCGGAGATTCGTAGCTGGA-3'. |
| IL6 | FP 5'-ACTCACCTCTTCAGAACGAATTG-3',  RP 5'-CCATCTTTGGAAGGTTCAGGTTG-3'. |
| CD40 | FP 5'-ACTGAAACGGAATGCCTTCCT-3',  RP 5'-CCTCACTCGTACAGTGCCA-3'. |
| PDCD1 | FP 5'-CCAGGATGGTTCTTAGACTCCC-3',  RP 5'-TTTAGCACGAAGCTCTCCGAT-3'. |
| ITGA5 | FP 5'-GGCTTCAACTTAGACGCGGAG-3',  RP 5'-TGGCTGGTATTAGCCTTGGGT-3'. |
| ITGAV | FP 5'-ATCTGTGAGGTCGAAACAGGA-3',  RP 5'-TGGAGCATACTCAACAGTCTTTG-3'. |
| ITGB3 | FP 5'-GTGACCTGAAGGAGAATCTGC-3',  RP 5'-CCGGAGTGCAATCCTCTGG-3'. |
| Trem2 | FP 5'-CTGGAACCGTCACCATCACTC-3',  RP 5'-CGAAACTCGATGACTCCTCGG-3'. |
| Gal3st4 | FP 5'-GGATCAACTGTTAAGTCTTGCCT-3',  RP 5'-AGAGCTGCTCCCAGATTTGTG-3'. |
| Ap1b1 | FP 5'-ACTATGGGCTGCATCCGAGT-3',  RP 5'-GAGGTCTTTAAGCGTGTCCAG-3'. |
| Sla | FP 5'-ATGGGGAATAGCATGAAATCCAC-3',  RP 5'-AGATGGGTAGTCAGTCAGCAC-3'. |
| Cybb | FP 5'-AGTGCGTGTTGCTCGACAA-3',  RP 5'-GCGGTGTGCAGTGCTATCAT-3'. |
| Cd53 | FP 5'-GTCTGTGGCTGTTGCATTTTG-3',  RP 5'-GAAGGGAAGGTTACGGAAGAGTA-3'. |
| Sla37a2 | FP 5'-CTTCGCGCCTTCTCTAGGG-3',  RP 5'-CATCTTTGTCGAATGGACTCCAG-3'. |
| Abi3 | FP 5'-CTACTGCGAGGATAACTACTTGC-3',  RP 5'-CAGGTTACCCACTTGGTAGGC-3'. |
| Rin3 | FP 5'-CACCCTGCCGGTCCTATTC-3',  RP 5'-CCTGGCCTAGACCTAGCTG-3'. |
| Scin | FP 5'-GAGGTTGACGTTGATGCAAATTC-3',  RP 5'-CTGGTTCCTTGCCTTCTTGAA-3'. |
| C3 | FP 5'-GAGCGAAGAGACCATCGTACT-3',  RP 5'-TCTTTAGGAAGTCTTGCACAGTG-3'. |
| Plekho2 | FP 5'-ATCCACGACTGCCCAAGGA-3',  RP 5'-GCACTAGCCCAGGGGTTTC-3'. |
| Plxdc2 | FP 5'-CCTGCTACTGGATGACGGAC-3',  RP 5'-TGCCGATGAGTGTTGGAAAGT-3'. |
| Cd163 | FP 5'-GGTGGACACAGAATGGTTCTTC-3',  RP 5'-CCAGGAGCGTTAGTGACAGC-3'. |
| Siglec10 | FP 5'-CTGCTGGGCCCCTCCTGC-3',  RP 5'-GACGTTCCAGGCCTCACAG-3'. |
| Gapdh | FP 5'-AGGTCGGTGTGAACGGATTTG-3',  RP 5'-GGGGTCGTTGATGGCAACA-3'. |
