# Supplementary material for: Interrogation of macrophage-related prognostic signatures reveals a potential immune-mediated therapy strategy by histone deacetylase inhibition in glioma
Source: Front Oncol. 2025 Jun 6;15:1554845. doi: 10.3389/fonc.2025.1554845 (PMC12179195; doi:10.3389/fonc.2025.1554845)

**Interrogation of Macrophage-Related Prognostic Signatures Reveals a Potential Immune-Mediated Therapy Strategy by Histone Deacetylase Inhibition in Glioma**

**Xisen Wang^1,2,3,†^, Xuya Wang^2,†^, Lei Chen^1,3,4,†^, Haozhe Ding^2^, Jikang Fan^3,5^, Jianshen Liang^2^, Yu Zhang^1,3^, Yiming Li^1,3^, Yiming Zhang^2^, Yaohua Li^1,3^, Shengping Yu^1,3^, Chen Zhang^1,3^, Tao Li^1,3,*^, Xuejun Yang^2,*^**

^1^Department of Neurosurgery, Tianjin Medical University General Hospital, Tianjin, China

^2^Department of Neurosurgery, Beijing Tsinghua Changgung Hospital, School of Clinical Medicine, Tsinghua University, Beijing, China

^3^Laboratory of Neuro-Oncology, Tianjin Neurological Institute, Tianjin, China

^4^Department of Neurosurgery, Tianjin First Central Hospital, Tianjin, China

^5^Department of Neurosurgery, Tianjin Huanhu Hospital, Tianjin, China

* Correspondence:

Tao Li

[litao@tmu.edu.cn](mailto:litao@tmu.edu.cn)

Xuejun Yang

[ydenny@126.com](mailto:ydenny@126.com)

†These authors have contributed equally to this work and share first authorship

# Supplementary Data

Supplementary Material should be uploaded separately on submission. Please include any supplementary data, figures and/or tables.

Supplementary material is not typeset so please ensure that all information is clearly presented, the appropriate caption is included in the file and not in the manuscript, and that the style conforms to the rest of the article.

# Supplementary Figures and Tables

## Supplementary Figures


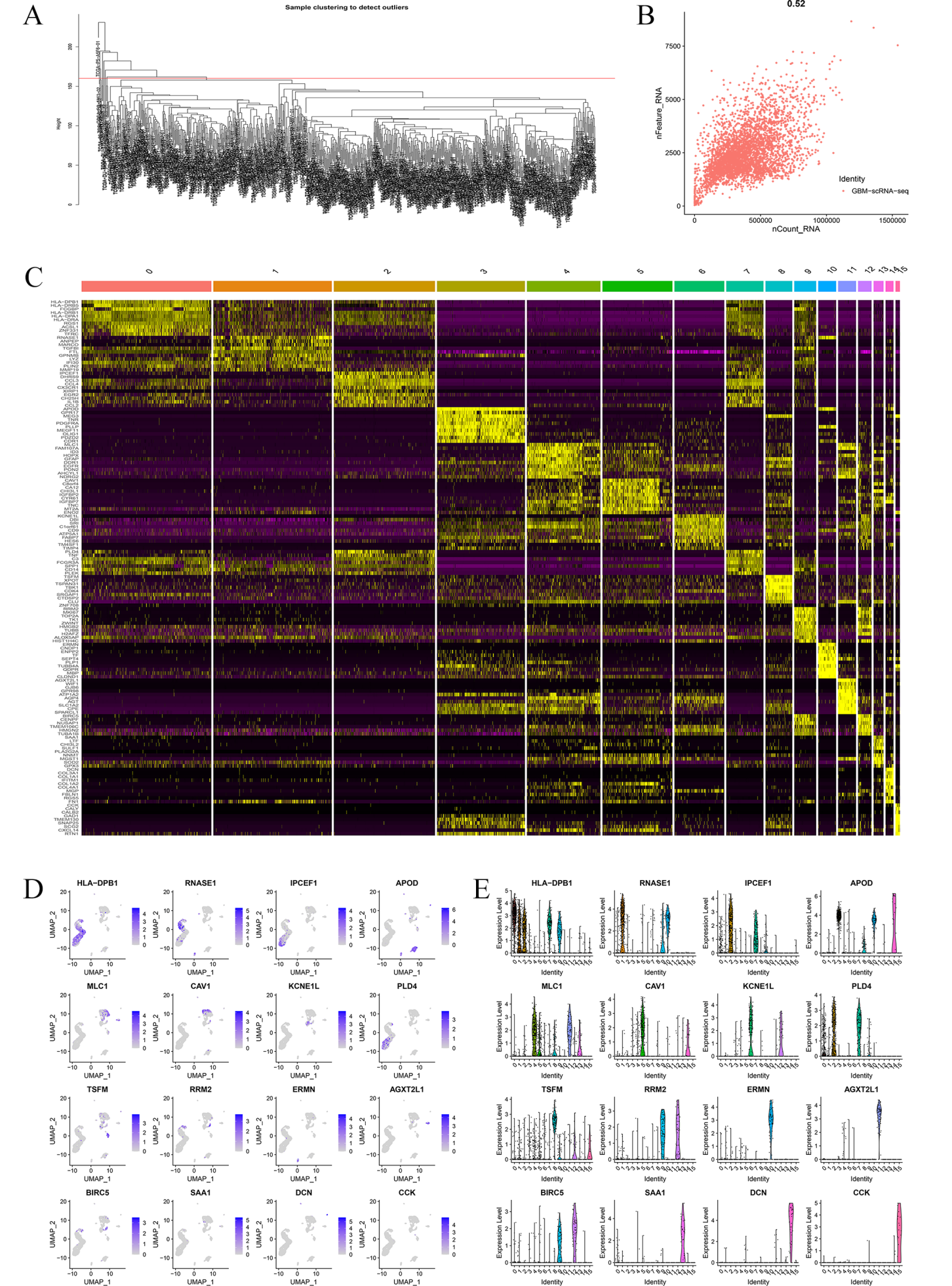


**Supplementary Figure 1.** (**A**) Clustering patients in TCGA using WGCNA and removing outliers. (**B**) Correlation between ncount and nfeature. (**C**) Heat map of the most significant differential genes for each of the 16 PCs. (**D**) The most significant differential genes of 16 PCs in single cells. (**E**) The most significant differential gene expression of the 16 PCs.

**
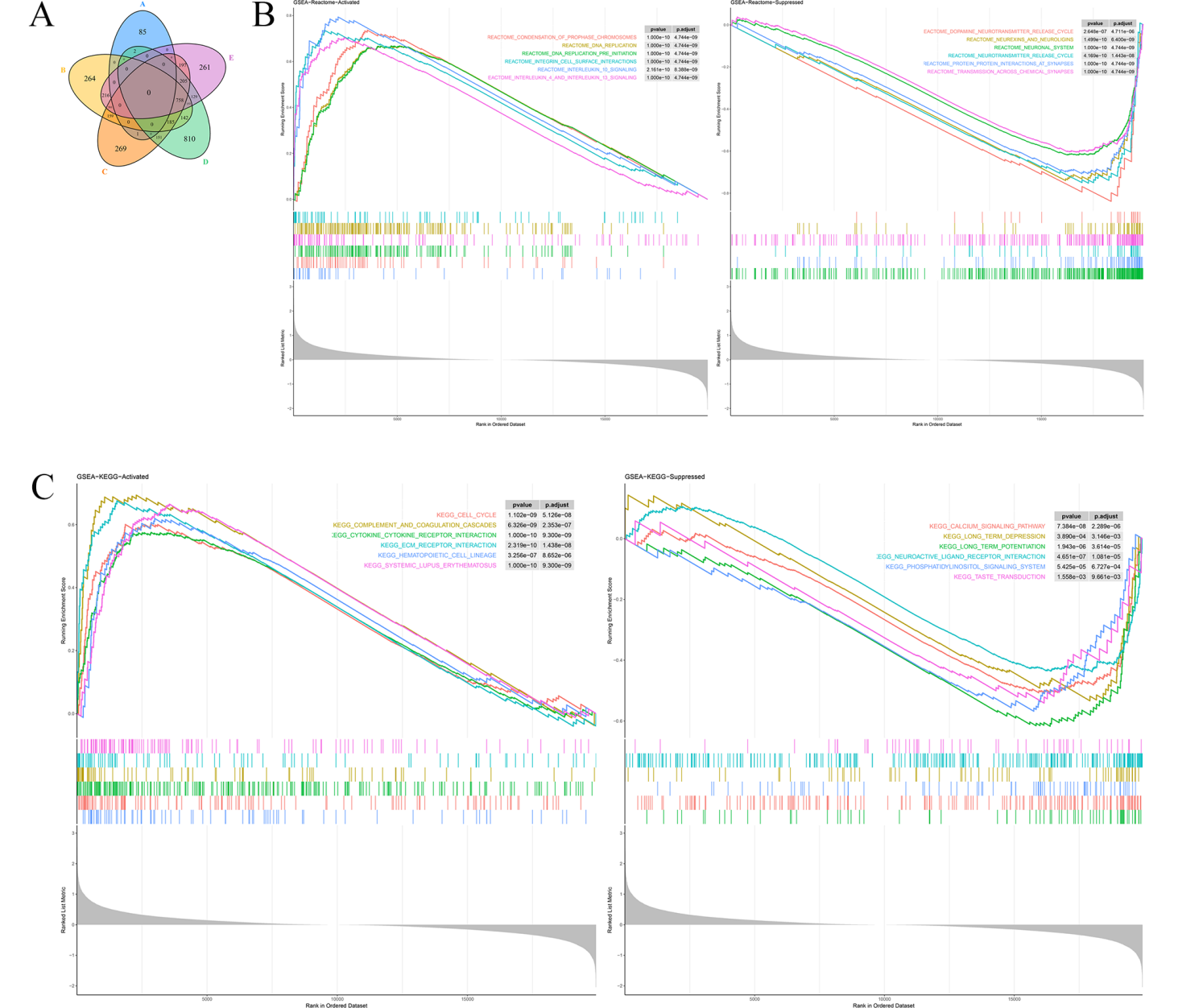
**

**Supplementary Figure 2.** (**A**) The intersection of MEsalmon gene set and 4 GAMs marker genes. (**B**) GSEA functional enrichment analysis of prognostic genes in Reactome database. (**C**) GSEA functional enrichment analysis of prognostic genes in KEGG database.


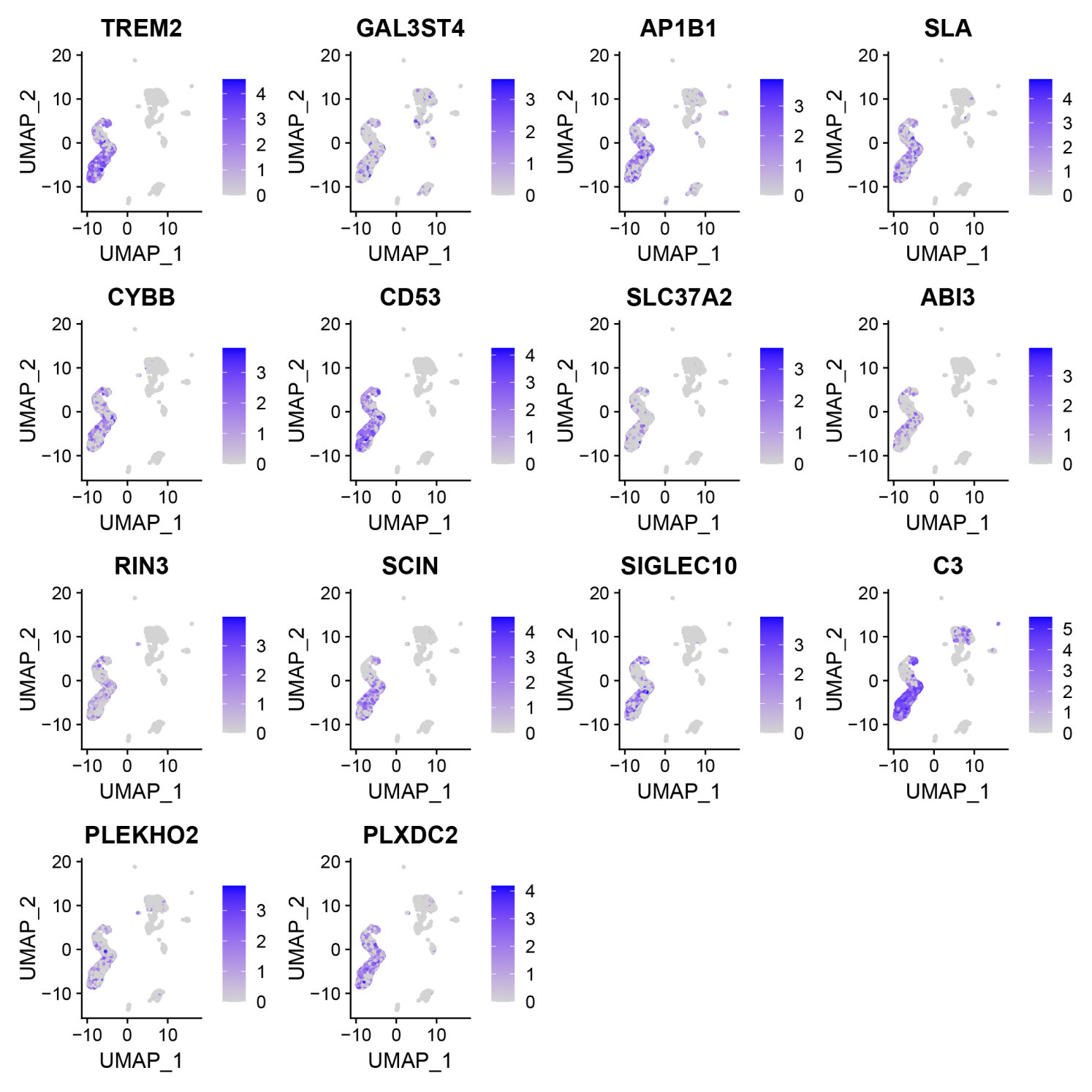


**Supplementary Figure 3.** Expression patterns of 14 prognostic genes in single-cell data.


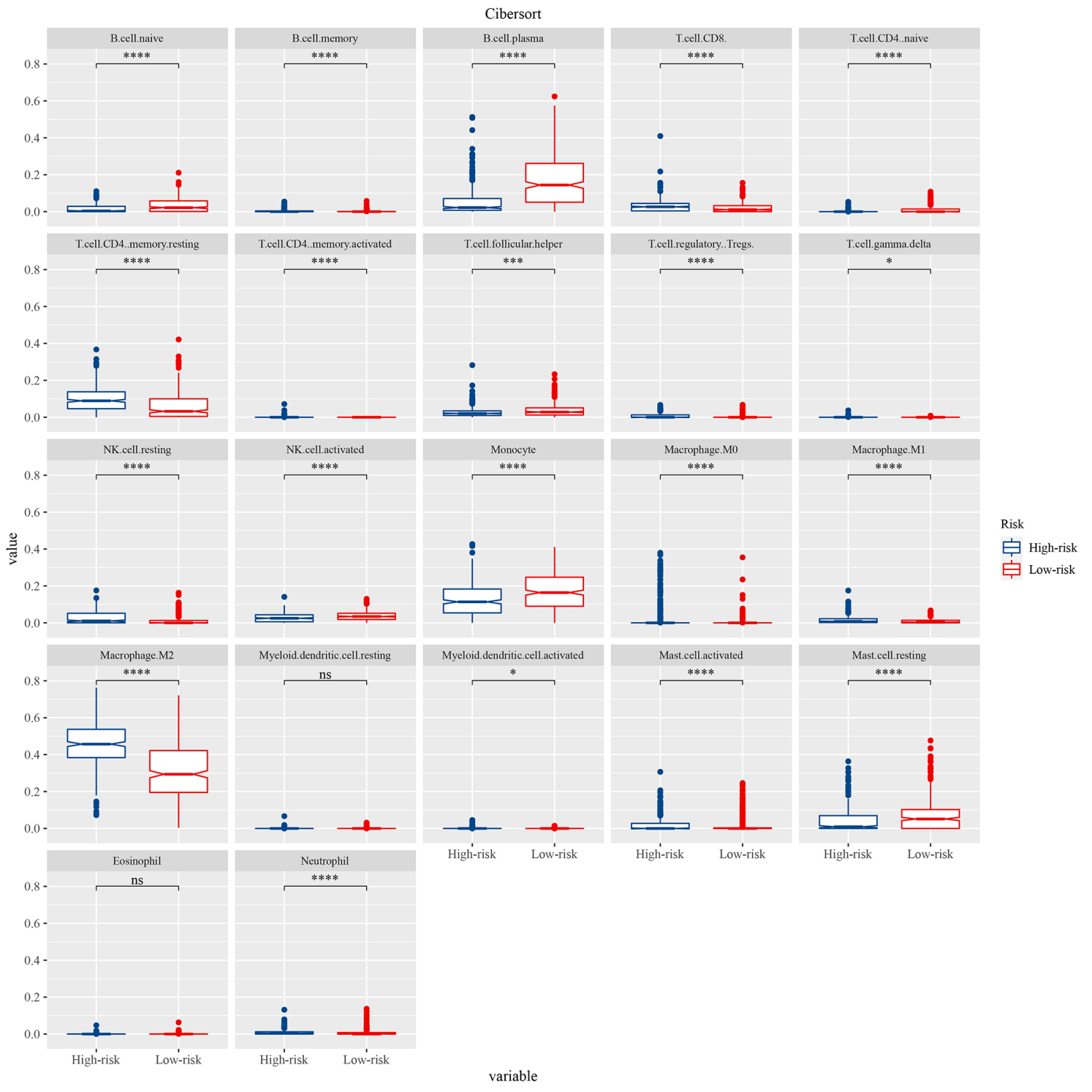


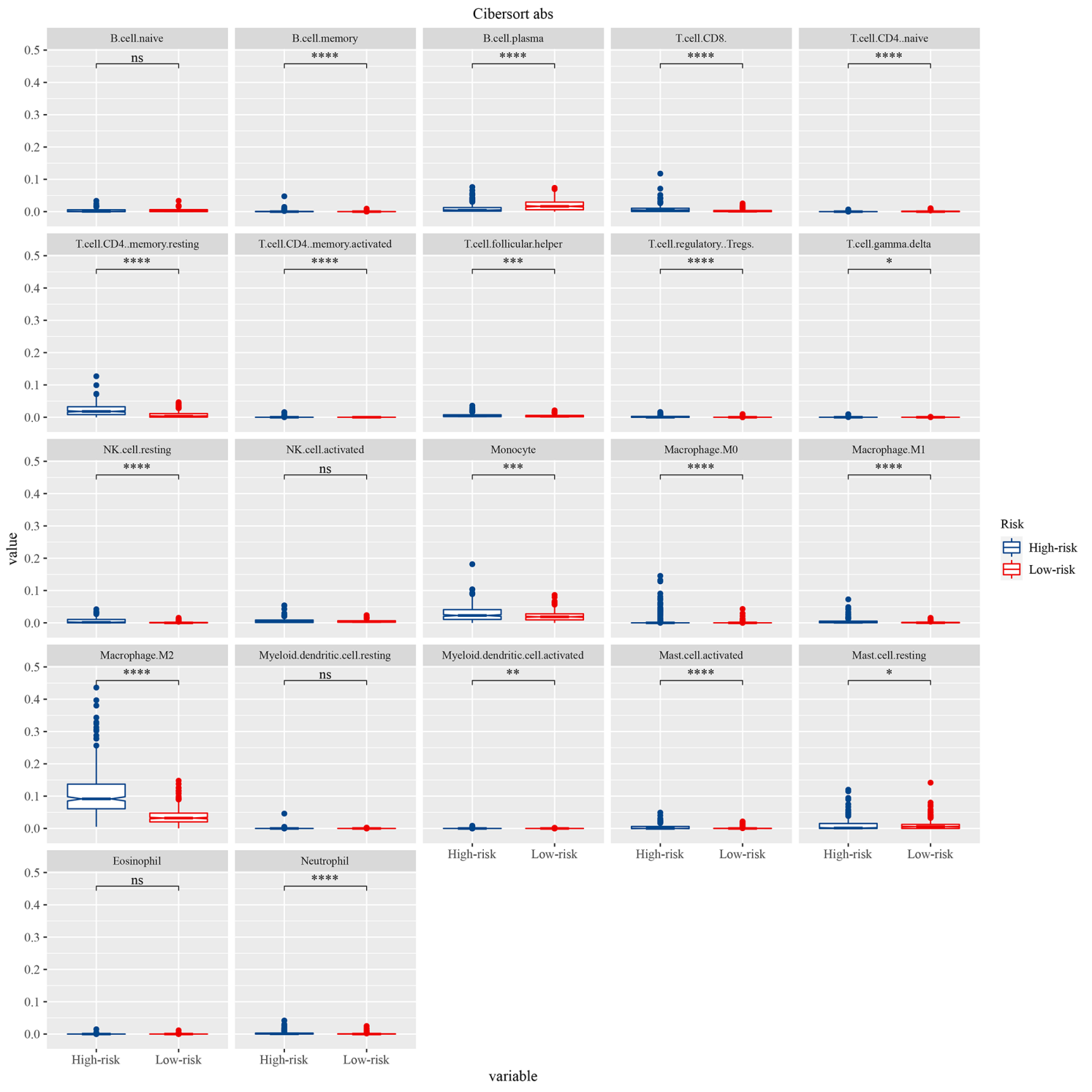


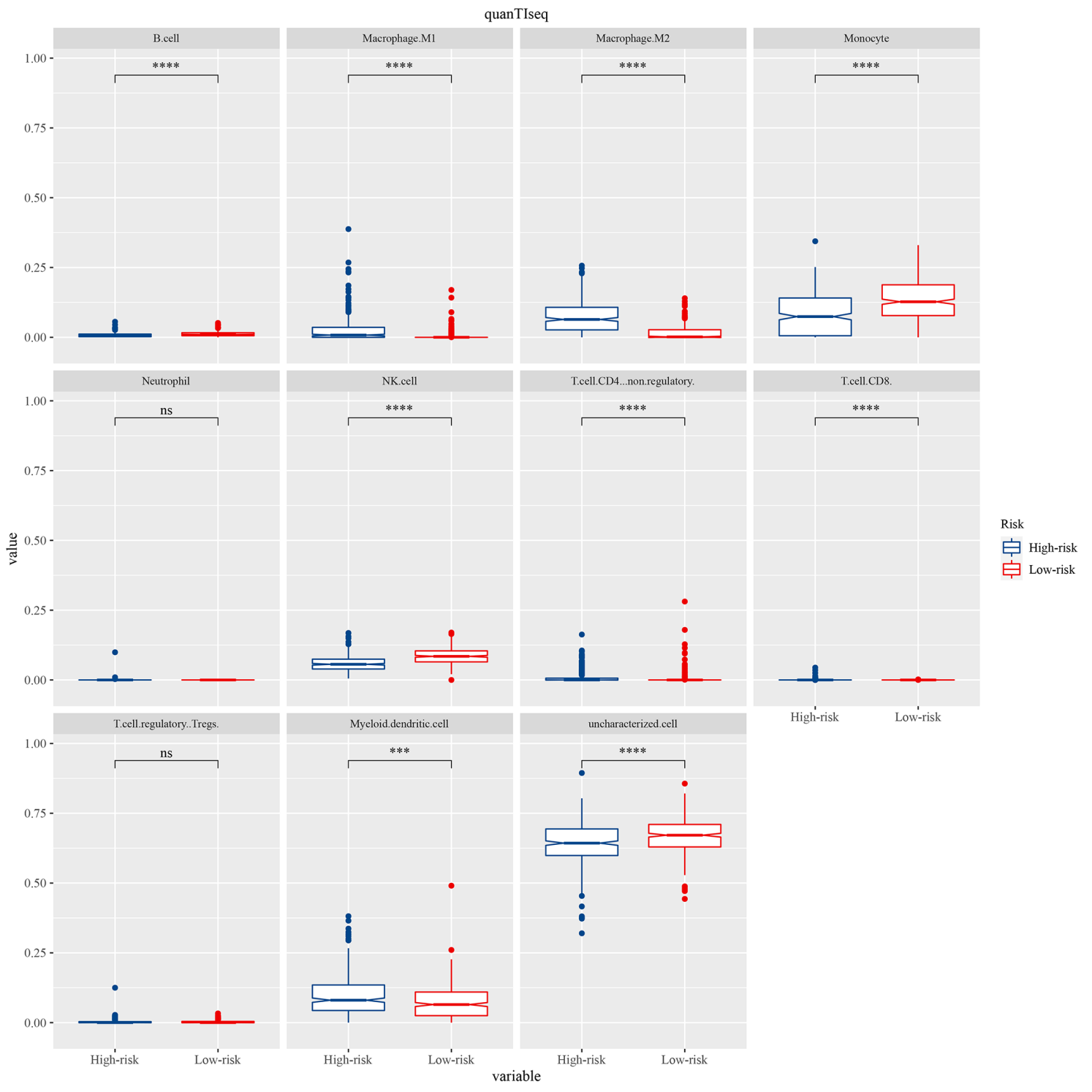


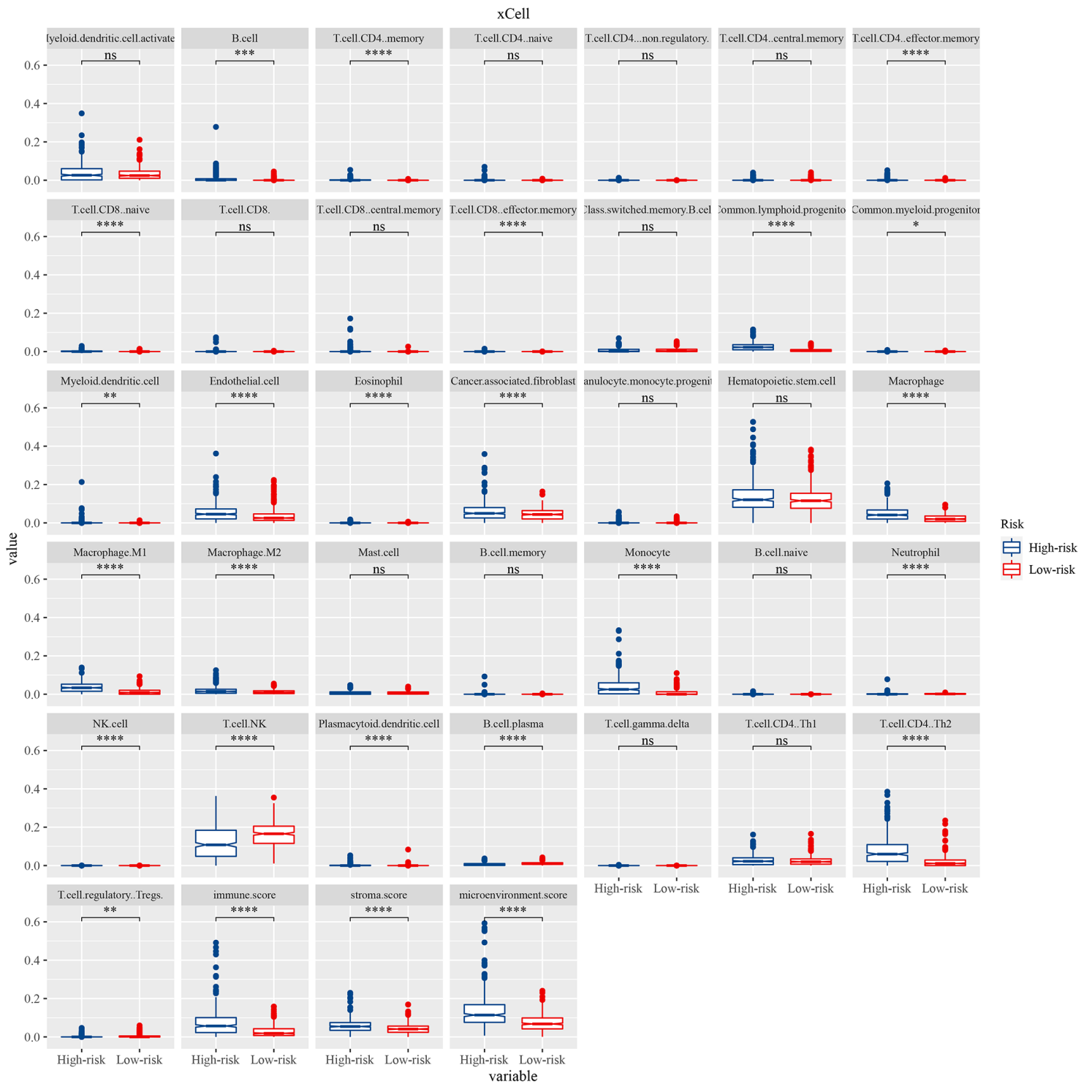


**Supplementary Figure 4.** Immune cell distribution in glioma microenvironment under four immune infiltration algorithms.


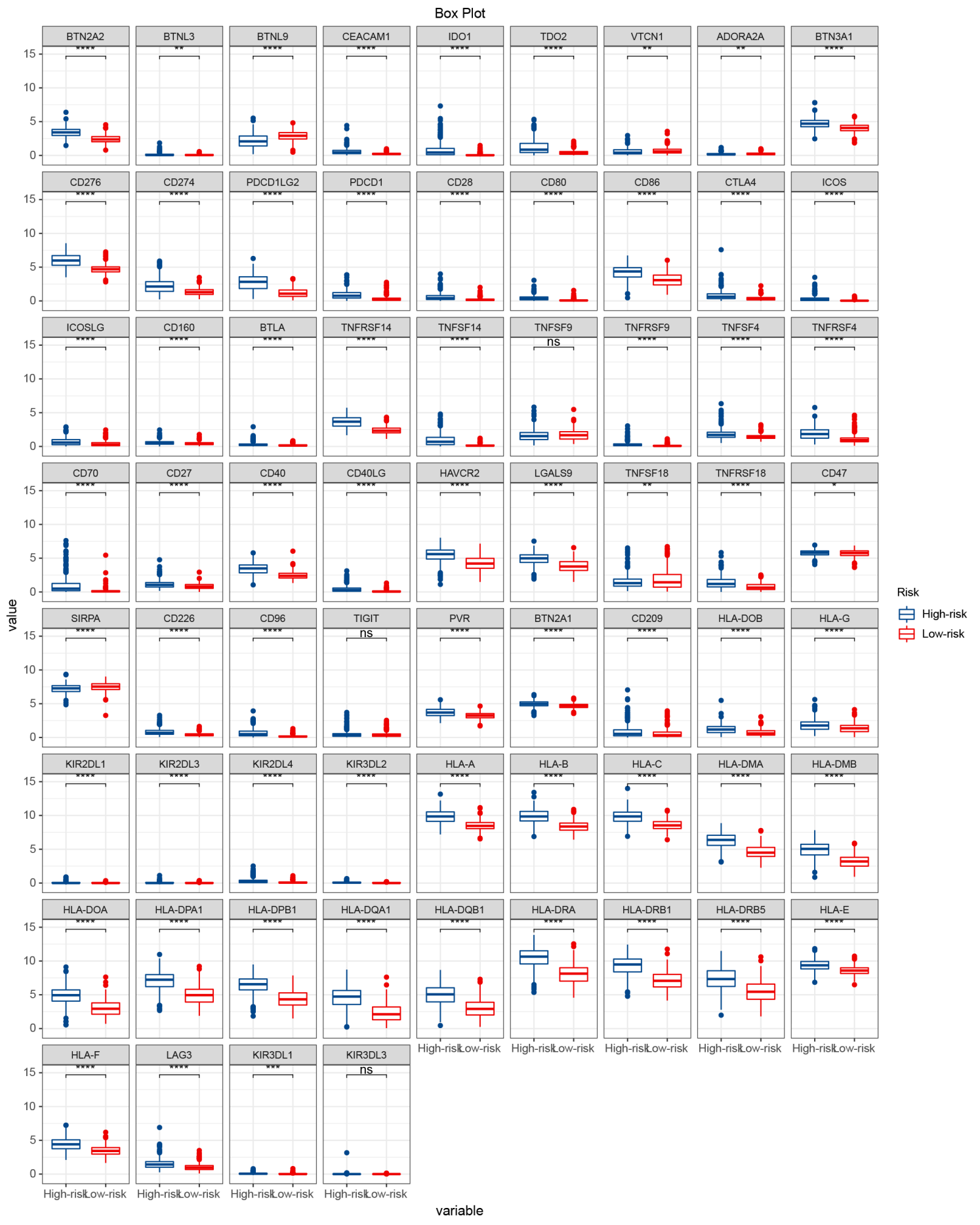


**Supplementary Figure 5.** Expression of immune checkpoint-related genes in high-risk and low-risk groups.


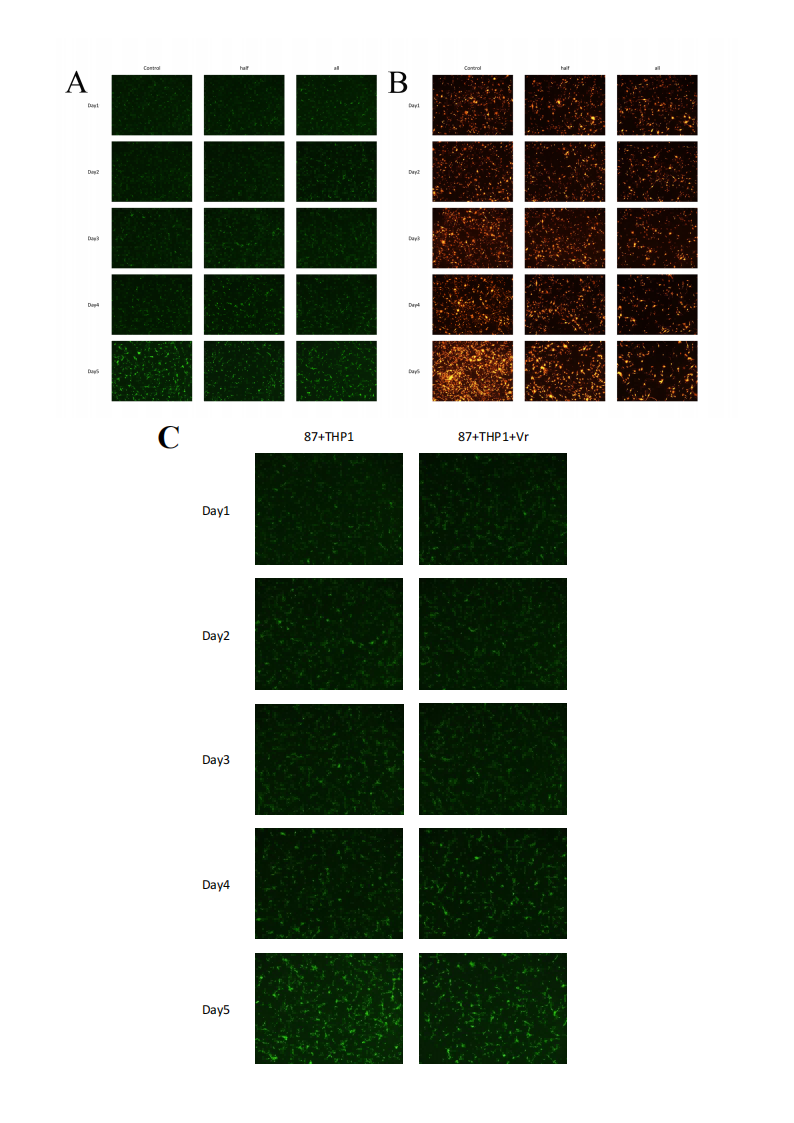


**Supplementary Figure 6.** (**A**) THP1(GF) under direct co-culture model. (**B**) U87(RF) under direct co-culture model. (**C**) THP1(GF) under the direct co-culture model of Vorinostat-treated and non-treated groups.

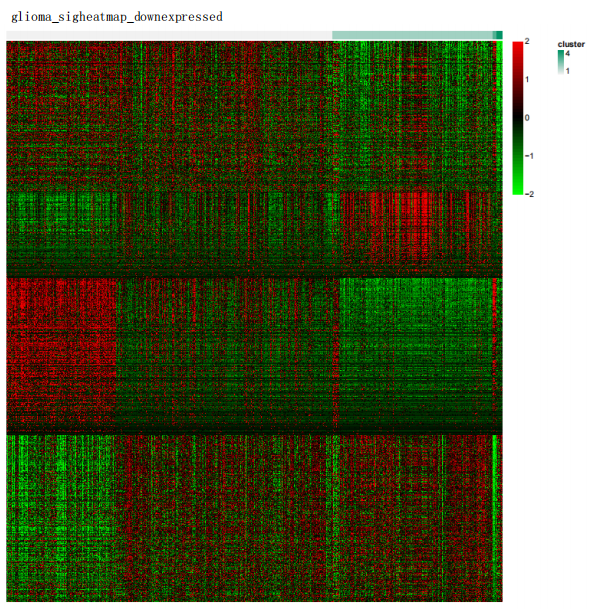

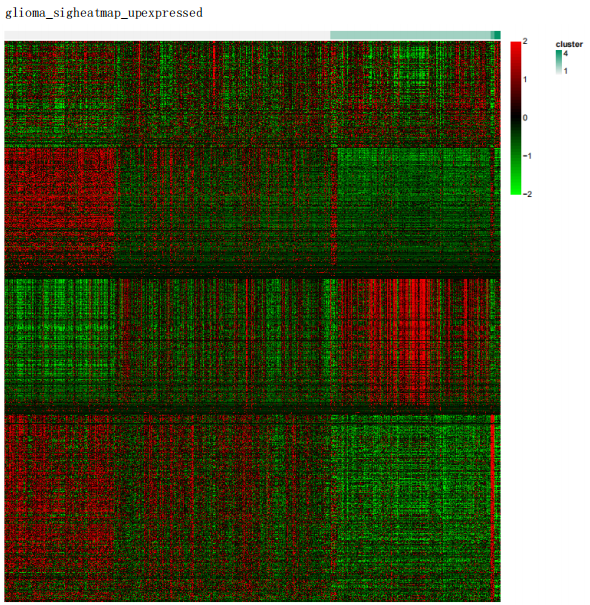


**Supplementary Figure 7.** Consensus clustering divided TCGA-GBMLGG into four groups, and the panorama of upregulated and downregulated genes among the four groups is shown.


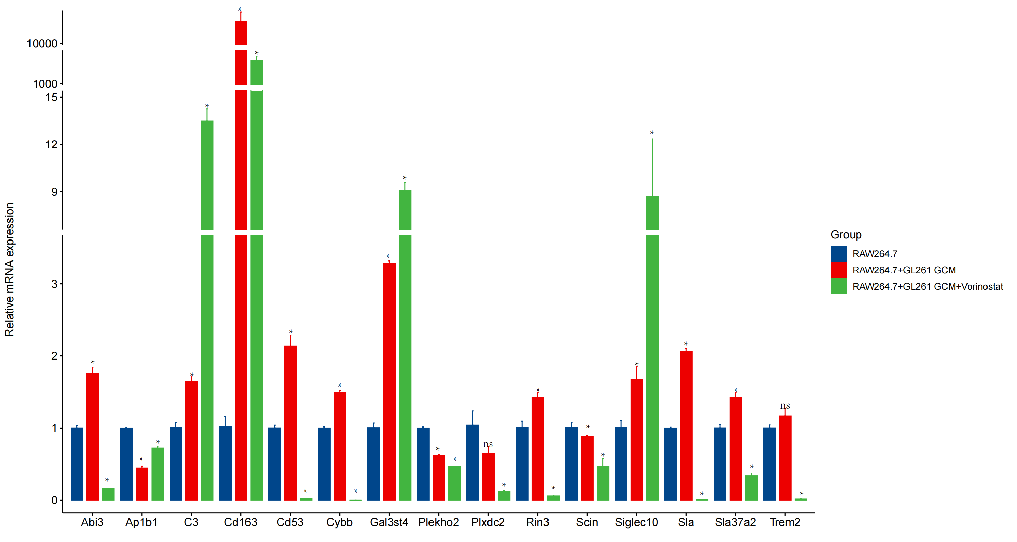


**Supplementary Figure 8.** The expression changes of 14 prognostic genes and *Cd163* in the mouse GL261-RAW264.7 co-culture model and after Vorinostat treatment.

Western Blot Original Image

Image 1
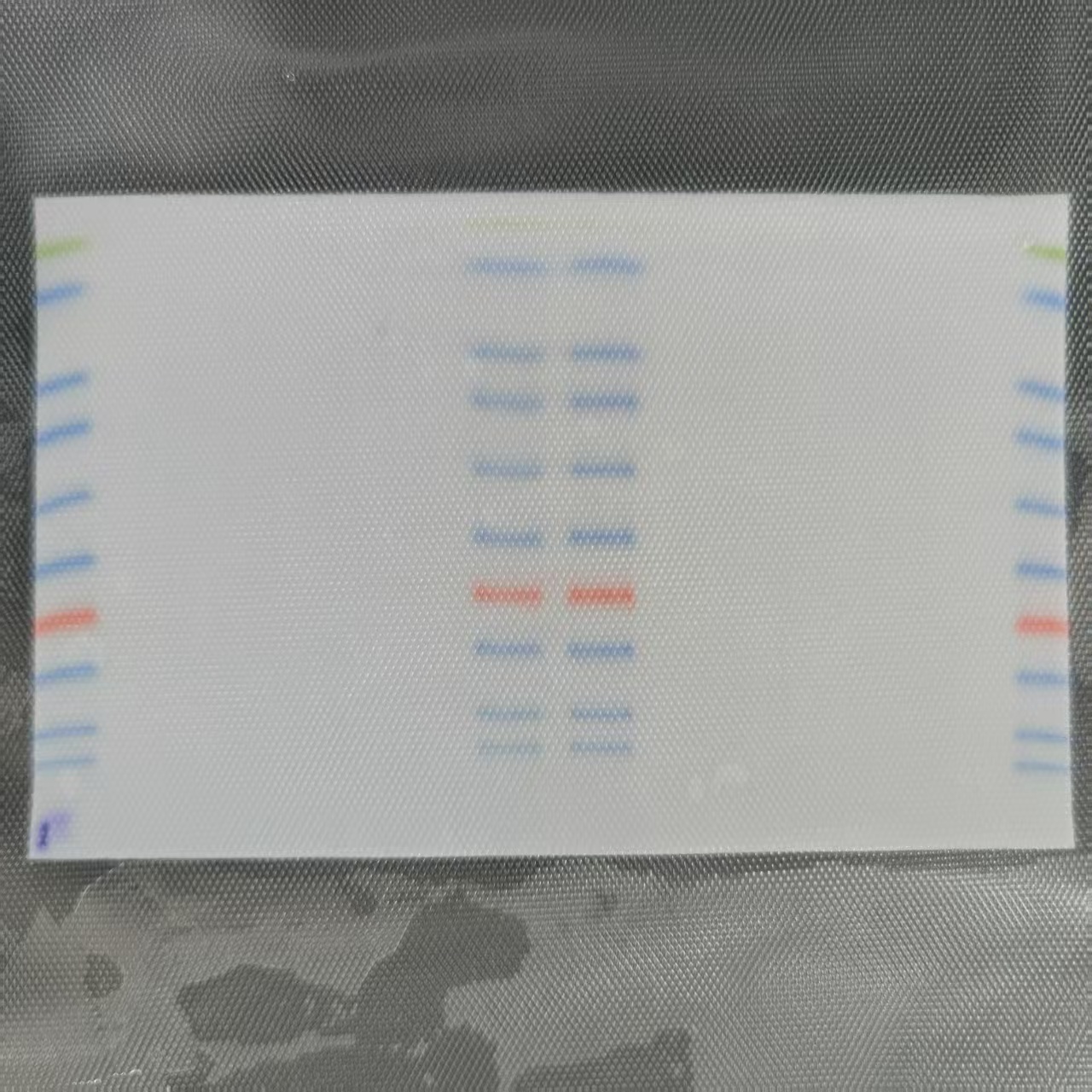


Cd163
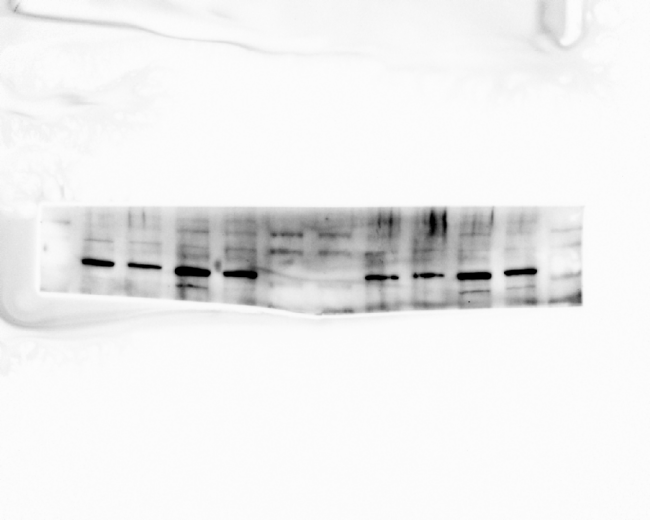


β-Tubulin
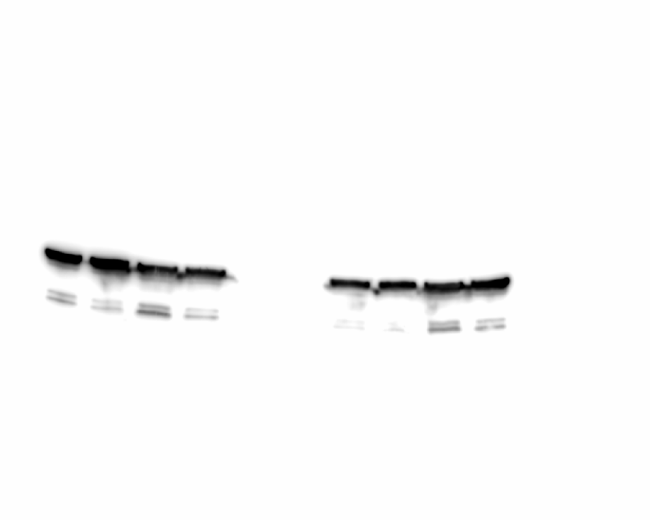


Image 2
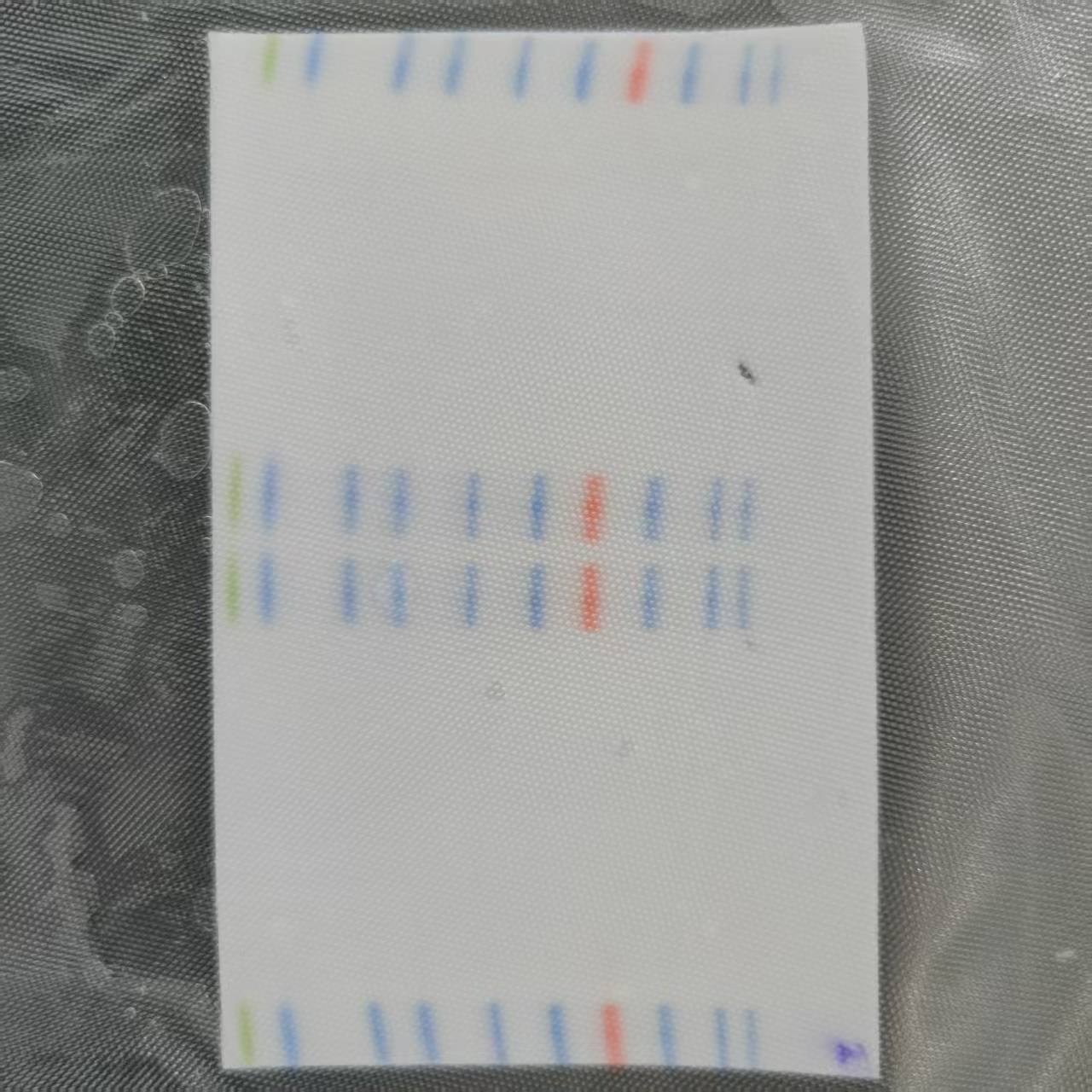


Cd163
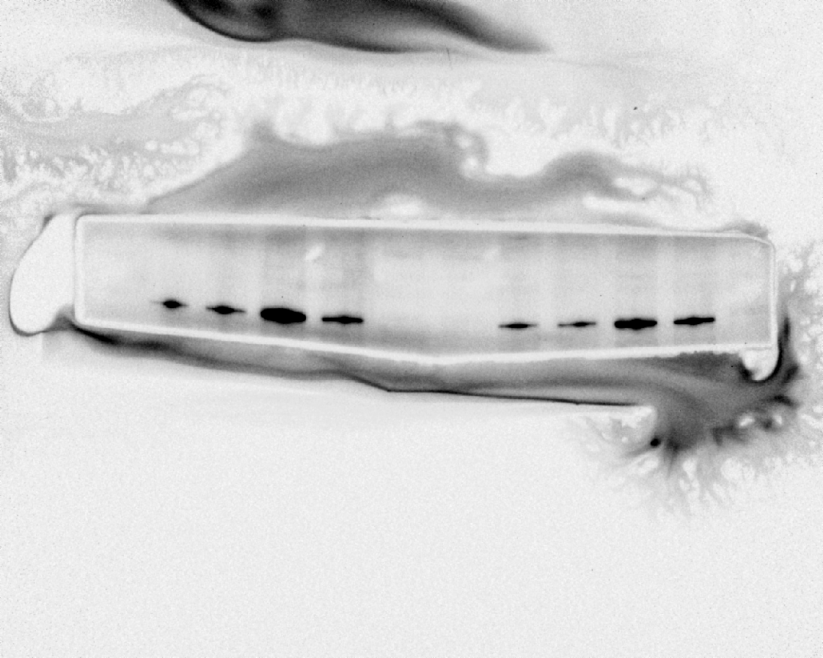


β-Tubulin
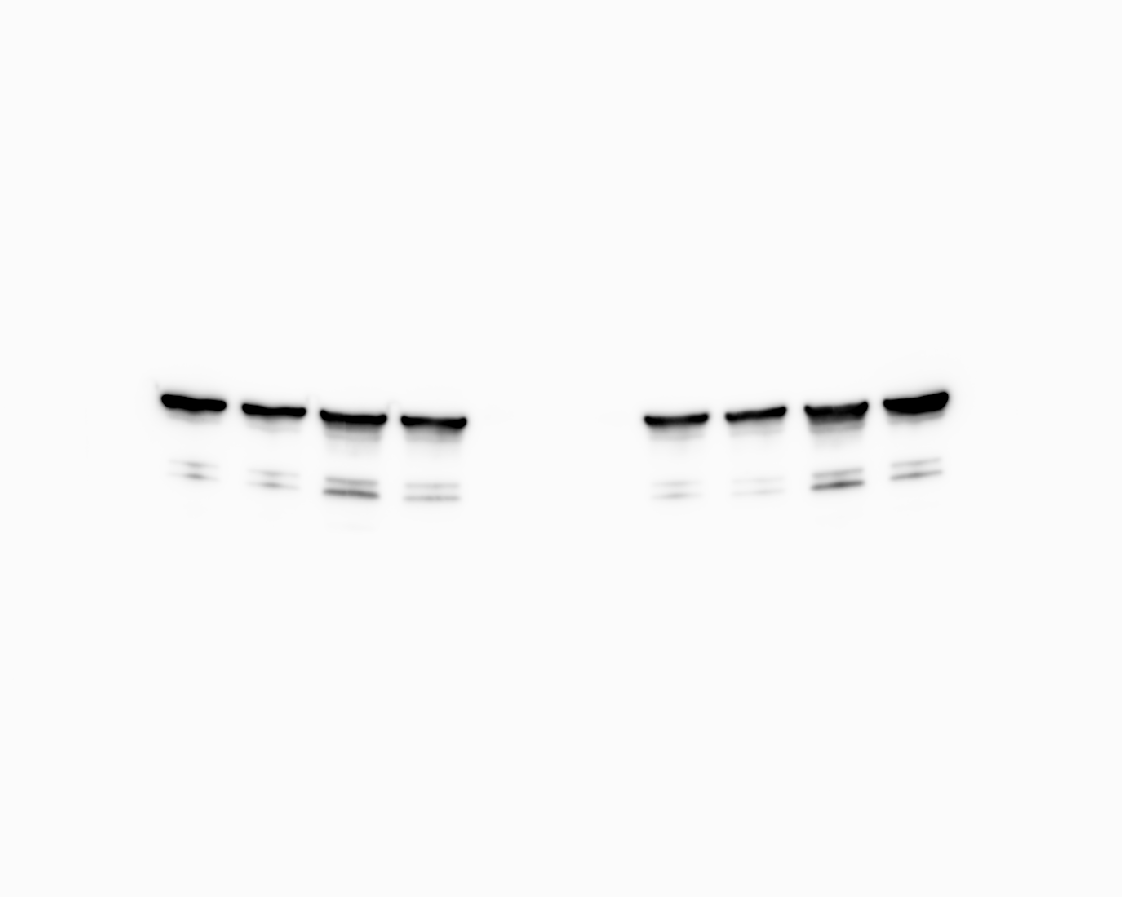

Supplement: Supplementary file 15 [file Table2.docx]
